# New insights on anti-tumor immunity of CD8^+^ T cells: cancer stem cells, tumor immune microenvironment and immunotherapy

**DOI:** 10.1186/s12967-025-06291-y

**Published:** 2025-03-17

**Authors:** Yibin Lin, Yifu Song, Yaochuan Zhang, Xiaodong Li, Liang Kan, Sheng Han

**Affiliations:** 1 Department of Neurosurgery, The First Hospital of China Medical University, Shenyang, 110001 China; 2 Department of Neurosurgery, Siping Central People’s Hospital, Siping, Jilin 136000 China; 3 Department of Geriatrics, Shengjing Hospital of China Medical University, Shenyang, 110004 China

**Keywords:** Cytotoxic CD8^+^ T lymphocyte (CTL), Cancer stem cell (CSC), Cancer prognosis, Tumor immune microenvironment (TIME), Immunotherapy

## Abstract

Recent breakthroughs in tumor immunotherapy have confirmed the capacity of the immune system to fight several cancers. The effective means of treating cancer involves accelerating the death of tumor cells and improving patient immunity. Dynamic changes in the tumor immune microenvironment alter the actual effects of anti-tumor drug production and may trigger favorable or unfavorable immune responses by modulating tumor-infiltrating lymphocytes. Notably, CD8^+^ T cells are one of the primary tumor-infiltrating immune cells that provide anti-tumor response. Tumor cells and tumor stem cells will resist or evade destruction through various mechanisms as CD8^+^ T cells exert their anti-tumor function. This paper reviews the research on the regulation of tumor development and prognosis by cancer stem cells that directly or indirectly alter the role of tumor-infiltrating CD8^+^ T cells. We also discuss related immunotherapy strategies.

## Introduction

The process of tumor immunity has been extensively investigated. Specific antigens are expressed by tumor cells with oncogenic mutations to distinguish between malignant and healthy cells as well as help immune cells, e.g., antigen-presenting cells (APCs), in identifying suitable tumor antigens. APCs then present tumor antigens to T cells in the lymph nodes and activate them. Activated T cells infiltrating into the tumor detect specific antigens on cancer cells and eventually kill them. During this process, several factors in the tumor microenvironment (TME) differentially regulate the activity and functional effects of tumor-infiltrating T cells.

Cytotoxic T cells, or CD8^+^ T cells detect tumor or infected cells by binding their T cell receptor (TCR), specifically to the MHC class I peptide complex. Effector CD8^+^ T cells develop into memory cells that halt re-exposure to the antigen after clearing. Effector CD8^+^ T lymphocytes become depleted in circumstances when the antigen is not eliminated, i.e., during cancer or persistent infections, conditions that reduce proliferation and cytotoxicity. Inhibitory molecules like PD-1 are expressed by depleted CD8^+^ T cells, which increase the depletion phenotype [1]. Immune checkpoint blockade (ICB) medications reduce inhibitory signaling in depleted CD8^+^ T lymphocytes to restore their proliferative and cytotoxic activities by targeting PD-1 and other inhibitory pathways [2].

Cancer stem cells (CSCs) were first identified in leukemia and then isolated in the 1990s via expression of CD34^+^ and CD38^−^ surface markers [3, 4]. CSCs can self-renew and differentiate into various cell subtypes. Besides, the activity of tumor-producing CSCs is modulated by several intracellular and extracellular factors acting as drug targets to treat cancer. In TME, CSCs promote tumor cell invasion and metastasis through several mechanisms, including enhanced angiogenesis and cytokine production [5]. Furthermore, the cytolysis activity of CD8^+^ T cells is suppressed by immunosuppressive cells infiltrating TME, including tumor-associated macrophages (TAMs), regulatory T cells (Tregs), and myeloid-derived suppressor cells (MDSCs); they secrete transforming growth factor-β (TGF-β), IL-6, and other cytokines [6].

This paper discusses the mechanism by which CSCs regulate anti-tumor immunity of tumor-infiltrating CD8^+^ T cells and the effect of CSCs on tumor progression and prognosis by regulating the role of CD8^+^ T cells. Moreover, we will investigate the role of immune microenvironment in these processes as well as their implications for immunotherapy.

## Basic mechanisms of CD8+ T cells

### Activation and infiltration

Common lymphoid progenitor cells develop in the red bone marrow and progress into immature precursor T lymphocytes. Thymic factor synthesis guides immature progenitor T cells (TCR- and CD-negative [double-negative]) into the thymus. The same chemicals stimulate the thymus’ production of TCR and CD proteins. Thymocytes detect and present CD- and TCR-positive T lymphocytes expressing MHC-1 and MHC-2 molecules. T cells with a high affinity for the body peptides suffer apoptosis via various ways to reduce the danger of an immunological response to peripheral self-proteins. T cells with TCR affinity for MHC-1 become CD8^+^ T cells, whereas T cells with TCR affinity for MHC-2 become CD4 + T cells.

**Fig. 1:**
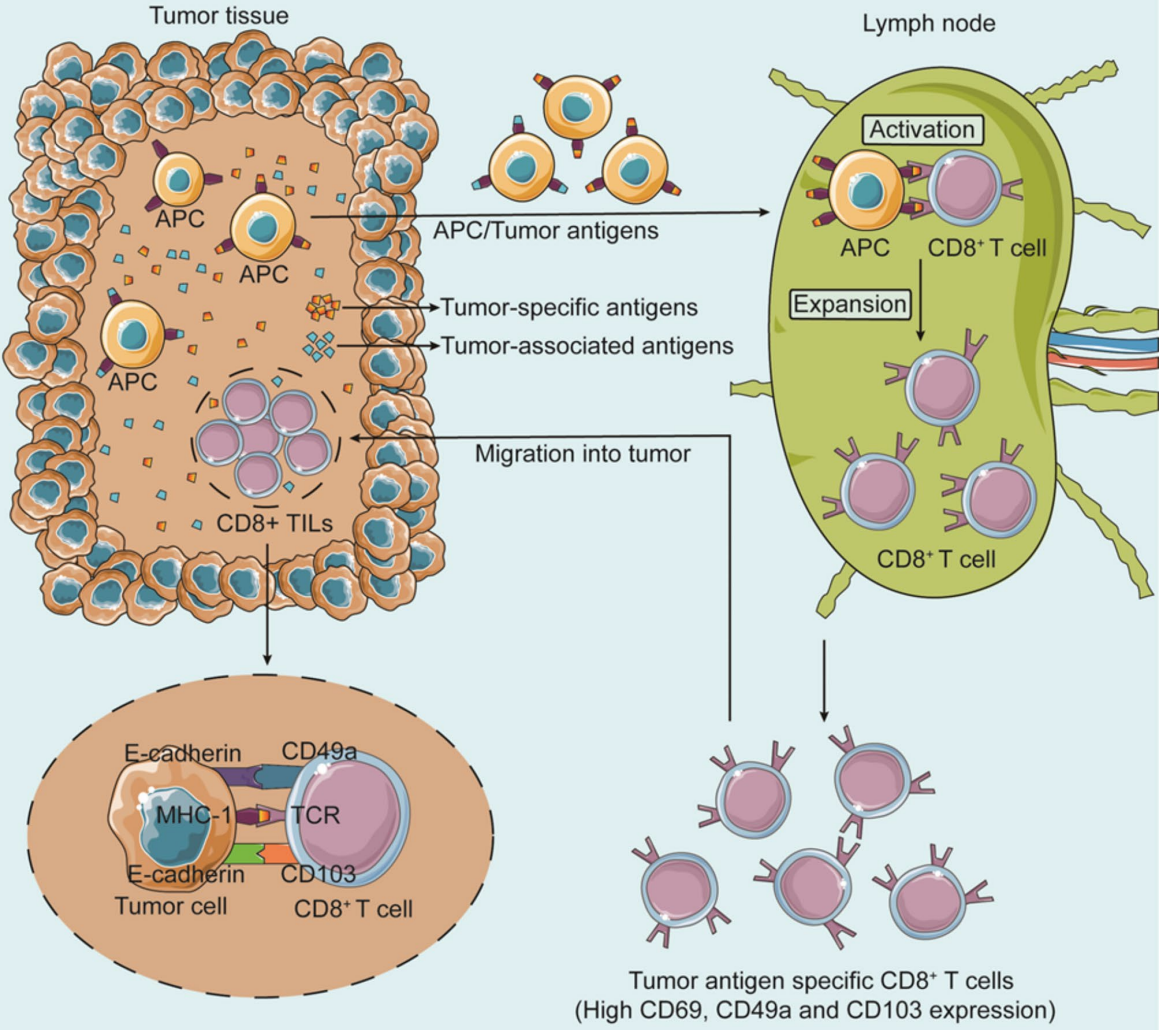
The process of CD8^+^ T cell infiltration into tumor tissue Tumor cells release tumor antigens (Tas) into the tumor microenvironment (TME), which include tumor-associated antigens (TAAs) and tumor-specific antigens (TSAs). In lymph nodes, antigen-presenting cells (APCs), such as dendritic cells (DCs), identify and bind to TAs. Tumor-infiltrating APCs carrying TAs then migrate to the lymph node, process and present TAs to CD8^+^ T cells, activating them and producing TA-specific CD8^+^ T cells. CD49a and CD103 are expressed on the surface of TA-specific CD8^+^ T cells and can bind to E-cadherin on tumor cells. TA-specific CD8^+^ T lymphocytes infiltrate tumor tissue, recognize TAs on tumor cells, and induce tumor cell death

Upon the first encounter of antigen-presenting cells (APCs) in secondary lymphoid organs (e.g., lymph nodes) that detect and bind tumor-specific antigens, naïve CD8^+^ T cells bind to the MHC-I of the APCs via their TCR/CD3 complexes [7]. Several signaling factors secreted by the APCs including (IL-12 and IL-2) [8] or IFN improve this stimulation and activate CD8^+^ T cells, thereby initiating rapid as well as extensive expansion and differentiation. The T cells then infiltrate into the tumor tissue, detect tumor antigens on the tumor cells, and bind to tumor cells (Fig. 1).

Dendritic cells (DC) are the strongest antigen-presenting cells first discovered by Steinman in 1973 [9]. Tumors can hinder the antitumor properties of DCs by disrupting their transport, preventing their maturation, and inducing differentiation of resistant subtypes [10]. Although the specific role of CSC in these processes is poorly understood, recent experimental results support the idea that CSC disrupts CD8^+^ T-cell activation, thereby suppressing anti-tumor immunity through similar mechanisms. GSCs inhibit DC maturation by downregulating miR-106a/20b levels and thereby upregulating STAT3 expression [11]. Furthermore, high FGL2 expression in GSCs inhibits CD103 + DC differentiation by suppressing NF-κB, STAT1/5, and p38 activation [12].

### Cytotoxicity

The persistent migration of CD8^+^ T cells on the target cells is a feature of T cell-target cell interactions. By secreting lethal particles with granzyme, perforin, histone C, and granulin that fuse with the target cell membrane or by absorbing the complex of granzyme, perforin, and granzyme via endocytosis of the cytotoxic T cell membrane, these mechanical forces increase pore formation in the target cell membrane ultimately killing the target cell. A few granzymes are released into the cytoplasm when the endosomal membrane becomes perforated. Additionally, Fas ligand (FASL) is expressed on CD8^+^ T cells and activates structural domains of death (Fas-associated protein with death domains [FADD]) by attaching Fas receptor on the target cell, which in turn activates cysteo asparaginase and endonuclease, resulting in target cell DNA breaks [13]. Ji et al. discovered that CSC suppressed CD8^+^ T cell cytotoxicity in triple-negative breast cancer, hence causing immunosuppression via the CHI3L1/MAF/CTLA4 axis [14].

### Differentiation and exhaustion

Due to chemokine production, aberrant tumor angiogenesis, and activation of the inhibitory checkpoint system, TME of solid tumors blocks CD8^+^ T cell transport and function. Upon tumor infiltration, naïve CD8^+^ T cells differentiate into effector CD8^+^ T cells. These cells then undergo further differentiation and activation to cytotoxic and memory CD8^+^ T cells, which exert specific activities at the tumor site. When cytotoxic CD8^+^ T cells first come into contact with foreign material, they typically generate cytotoxic cytokines and mediate tumor destruction tasks. After an initial encounter with the antigen, memory CD8^+^ T cells stay in a different region to perform their specific function. The development of the antigenic peptide-major histocompatibility complex (MHC) is important for CD8^+^ T cell differentiation. In addition, cytokine synthesis and co-stimulatory signals from antigen-presenting cells (APCs), extracellular cytokine secretion, and transcription, metabolism, and epigenetic factors influence differentiation.

Differentiation of CD8^+^ T cells is tightly controlled. T cell activation and differentiation processes are significantly disrupted by variations in the type, context, and length of antigen interactions. This may result in malfunctioning, unresponsiveness, and even death of T cells. Due to altered activation and differentiation processes, multiple phases of T cell dysfunction have been identified. Terms including fatigue, tolerance, anergy, ignorance, and senescence have been used to describe varying degrees of CD8^+^ T cell failure [15].

**Fig. 2:**
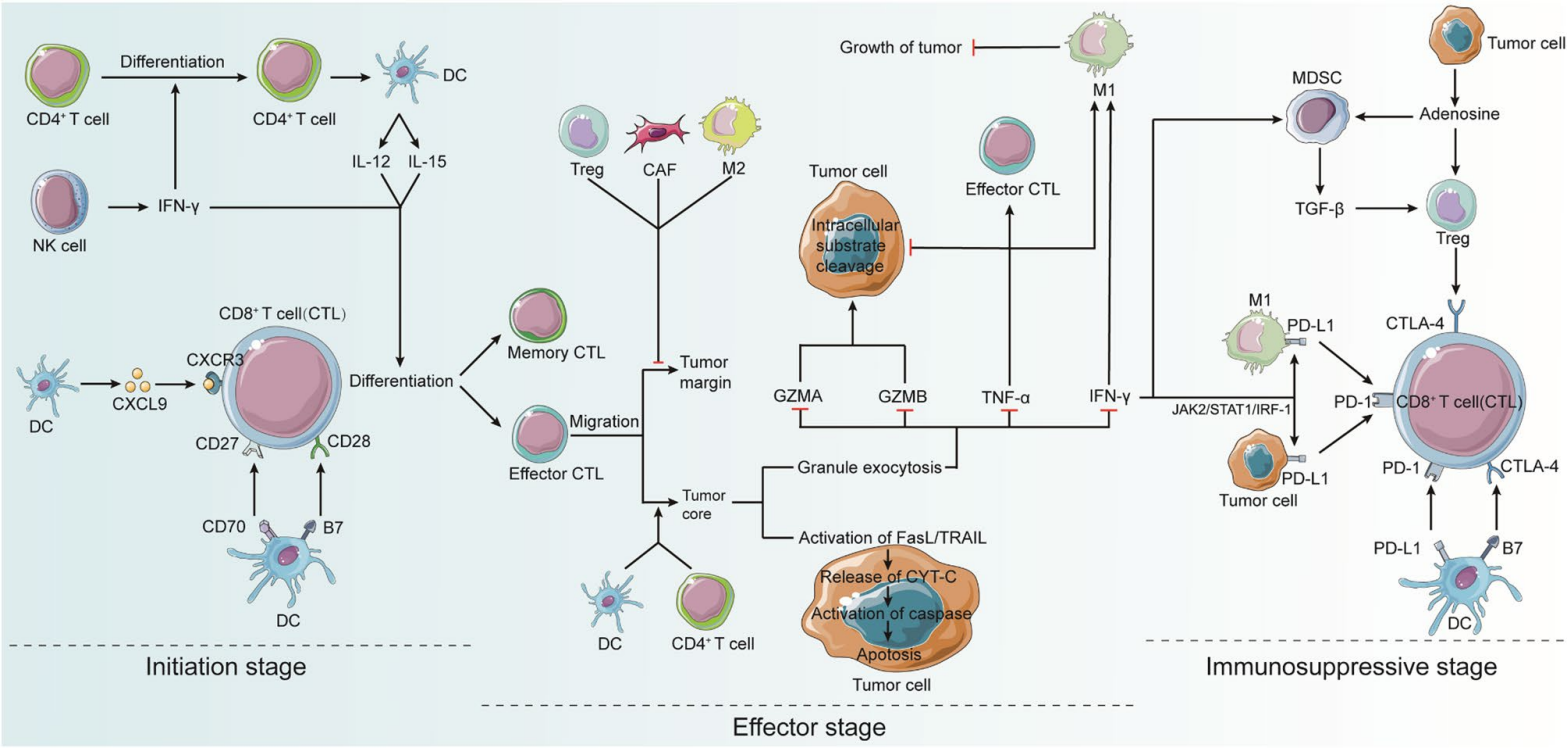
Activation and deactivation of CD8^+^ T cells Dendritic cells (DCs), natural killer (NK) cells, and CD4+ T cells play significant roles in the activation of CD8^+^ T cells. DCs interact with CD8^+^ T lymphocytes via receptor ligands. CD4^+^ T cells facilitates the activation of CD8^+^ T cells by stimulating them. DCs promote the development of CD4^+^ T cells into antigenspecific effector T cells. CD4^+^ T cells stimulate CD8^+^ T cell start via cytokines. CD4^+^ T cells can also contribute to DC activation and licensing by inducing DC maturation, co-stimulatory molecule expression, and cytokine secretion, all of which activate CD8^+^ T cells. NK cells also perform similar functions. In the effector stage, CTLs are activated to kill target cells by granule cytotoxicity and Fas ligand (FasL)-mediated apoptosis. CTLs emit IFN-γ and TNF-α, causing cytotoxicity in cancer cells. NK cells play similar functions. In the immunosuppressive phase, activated CTLs are activated and licensed to express co-stimulatory molecules and secrete cytokines. In the immunosuppressive stage, activated T cells begin to express co-inhibitory receptors, such as the programmed death-1 receptor (PD-1), within hours or days of activation. This occurs through IFN-γ induction of programmed death- 1 ligand (PD-L1) expression in anti-tumor M1 macrophages and cancer cells. Expression of CTL-associated antigen 4 (CTLA-4) by regulatory T cells (Tregs) can also inhibit the suppressive activity of CD8^+^ T cells, thereby triggering immunosuppressive activity within the TME

The dysfunctional condition called T cell exhaustion, in which simple antigen removal fails to restore CD8^+^ T cells, is defined and may be driven by the ongoing expression of immunological checkpoint molecules produced by CD8^+^ T cells exposed to tumor neoantigens. Within weeks of antigen exposure, CD8^+^ T cells continuously release more cytotoxic T lymphocyte-associated protein (CTLA-4), causing cell depletion and apoptosis. Depleted CD8^+^ T cells also continue to exert mitogenic activity, which promotes the establishment or preservation of a suppressive environment. Depleted T cells express inhibitory receptors, such as PD-1, LAG-3, Tim-3, 2B4/CD244, CD160, and TIGIT, which play a major role in regulating T cell function. In vivo blockade of PD-1 inhibitory receptors activates depleted T cell responses and enhances viral control, an important improvement in the field [2] (Fig. 2).

### Tolerance

Self-tolerance refers to the low response state of autoantigen-reactive T cells, which is necessary to prevent autoimmunity. Tolerance is mediated through two major ways, i.e., cerebral and peripheral. When naive, self-reactive T cells come into contact with self-antigens on non-activated or non-specialized antigen-presenting cells (APCs) in normal tissues; they cannot fully acquire initiation and activation signals in a non-stimulating environment. This can induce either apoptosis or the development of cellular intrinsic self-tolerance programs [16, 17]. Tumor cells can decrease antigen presentation and escape immune identification by inhibiting DC activity, disrupting antigen processing, and down-regulating production of HLA-1 by tumor cells [18]. The inability of CD8^+^ T lymphocytes to eradicate malignancies can be attributed to several factors including T cell-extrinsic mechanisms, cancer cell-mediated processes (such as loss of MHC expression, antigen loss, loss or abnormalities in antigen presentation, or expression of inhibitory receptor ligands) and TME-mediated mechanisms (including TGF-β, IL-10, nitrogen metabolites, regulatory T cells, or MDSCs) [19].

CTLs release cytotoxic molecules that cause substantial damage within tumor cells; however, tumors also develop various defense mechanisms to counter CTL attacks [20]. Generally, defensive mechanisms may be separated into two groups, i.e., constitutive and induced defenses. Two categories exist within the induced defense, i.e., rapid and slow mechanisms. The major functions of the fast defense system include counteracting cytotoxic chemicals in immunological synapses, initiating membrane repair pathways to close perforations in the membrane, and restricting the amount of granzyme entering the cell. Tumor-derived lysosomal cathepsin released into immune synapses degrades soluble perforin, restricting the inflow of cytotoxic molecules. This mechanism, which CTLs use to protect themselves from cytotoxic molecular damage, is mimicked by tumor cells. Besides the quick synaptic defense systems, delayed defense mechanisms also start within minutes to hours, trying to restore homeostasis, remove injured organelles, and accelerate recovery from non-fatal CTL assaults. For instance, autophagy induction (degradation of damaged organelles) promotes the survival of tumor cells in response to CTL attack, mediating resistance of melanoma cells to CTL attack; inhibition of autophagy (genetic or drug) can also render tumor cells more sensitive to CTLs attack. Constitutive defense mechanisms include regulated cell death (RCD) mutation and incapacitation, membrane component modification, as well as abnormal autophagy.

## CD8+ T cells and cancer stem cells in tumor development and prognosis

According to a recent study on immune cell subsets that infiltrate tumors, CD8^+^ T cells were found to boost patient survival. The positive predictive significance of CD8^+^ T cells was confirmed in 18,700 patients with 17 distinct forms of solid cancer [21]. This review examined the involvement of CD8^+^ T cells in tumors such as neurologic tumors [22–24], melanomas [25–28], squamous cell carcinoma [29–31], hepatocellular carcinoma [32, 33], colorectal carcinoma [34–36], lung carcinoma [37, 38], breast carcinoma [39, 40]. We also delved into other recent reports on the progression of various tumors [41]. Additionally, we discussed the role of CSC in regulating CD8^+^T cell anti-tumor immunity by interacting with these cells.

### Neurological tumors

Investigations on the immune microenvironment of intracranial tumors indicate that CD8^+^ TILs play a key role in TME of brain tumors [22]. Gliomas are the most prevalent primary malignant tumors of the central nervous system (CNS), with a high mortality and aggressiveness. In gliomas, low numbers of CD8^+^ TILs are associated with a poor prognosis [23, 24]. Our previous findings revealed upregulated levels of CD4 + TILs and downregulated levels of CD8^+^ TILs in high-grade gliomas compared to low-grade gliomas, which are independently associated with shorter progression-free survival (PFS) and overall survival (OS) in GBM [24]. Regulation of the CD8^+^ /CD4^+^ TIL ratio may have treatment implications for gliomas. However, additional studies are necessary to clarify these issues. CD8^+^ TILs play an equally critical role in some benign tumors of the nervous system. Meningiomas are among the most common primary cerebral tumors, most of which are benign. Our previous study reported peritumoral brain edema (PTBE) as an independent predictor of CD8^+^ TIL levels in meningiomas. Besides, CD8^+^ TIL levels were associated with meningioma recurrence [42]. Monitoring the level of CD8^+^ T-cell infiltration will help in assessing the prognosis of patients with meningiomas. Pituitary adenomas (PAs) are tumors of adeno-pituitary origin and the 3rd most common intracranial tumor. Additionally, CD8^+^ TIL levels are an independent predictive predictor for the recurrence of pediatric and adolescent pituitary adenomas (PAPAs) [43]. This highlights the significance of CD8^+^ TIL levels in TME and their effect on the evolution of PAPAs. Nevertheless, additional investigations are required to identify the exact molecular processes.

A recent comprehensive investigation of human tumor samples found that high CD133 expression in glioblastomas is responsible for recurrence [44]. xperimental findings by Ji, et al., further confirmed the suitability of CD133 as a CTL target for GSC immunotherapy. These results have prompted a phase I study of a dendritic cell vaccination using these CTL epitopes in recurrent glioblastoma [45]. The endothelial differentiation function of CSCs in glioblastoma promotes tumor vascularization and angiogenesis [46]. Additionally, differences exist in the degree of influence of different subtypes of CSCs on the infiltration of immune cells including CD8^+^ T cells in GBM. Beier et al. found that CSC subtypes modulate the level of immunological infiltration and local immune paralysis through variable degrees of TGF-β-mediated inhibition of invading immune cells [47]. G9a promotes activation of Notch signaling by suppressing Fbxw7 expression in GSCs, upregulates PD-L1 expression, and inhibits cytotoxicity in CD8^+^ T cells [48]. G9a may be a potential target for anti-tumor immunotherapy targeting GSCs. Xuan et al. showed that inhibition of the CLOCK-OLFML3-HIF1a-LGMN-CD162 axis reduces immunosuppressive microglia and increases CD8^+^ T cell infiltration, activation, and cytotoxicity within GBM, as well as creates a synergy with anti-PD-1 therapy [49]. Additionally, GSCs cause intracellular accumulation of crotonyl-CoA and histone H4 lysine crotonylation by reprogramming lysine catabolism. Type I interferon signaling is improved when histone lysine crotonylation is reduced via genetic modification or lysine restriction. This causes decreased GSC tumorigenic potential and increased CD8^+^ T cell infiltration [50].

### Melanoma

Previous studies have shown that CD8^+^ T-cell density, PD-L1, and expression of immune-related genes are biomarkers of melanoma prognosis [27, 28]. ZEB1, an epithelial-mesenchymal transition (EMT)-inducing transcription factor, has recently been discovered to be expressed in melanoma cells in association with a decrease in CD8^+^ T-cell infiltration independent of β-catenin pathway activation [51]. Targeting ZEB1 may be a promising strategy that promotes CD8^+^ T cell recruitment within tumors and increases immunotherapeutic responses in melanoma. Unlike healthy controls, patients with liver metastases from uveal melanoma exhibited higher percentages of inflammatory and immunosuppressive immune cells, and their DCs and CD8^+^ T cells displayed impaired activation. Nonetheless, following isolated hepatic perfusion (IHP), patients with adequate non-M2 macrophages and tumor-infiltrating CD8^+^ T cells had a better prognosis [52]. This suggests that the immune system may have a role in the efficacy of IHP with melphalan, and additional investigations on IHP in conjunction with immunotherapy is essential. Research on the role of CD8^+^ TILs in melanoma may improve knowledge of the immunological mechanisms underlying melanoma regression, which may promote the discovery of novel prognostic indicators to support clinical decision.

In melanoma, a vaccine for CSCs developed by Yin et al. et al. promotes DC maturation, activates CD8^+^ T cells, inhibits the expression of CTLA-4, PD-1, and Tim-3, as well as upregulates the expression of IFN-γ and GzmB in CD8^+^ T lymphocytes. The specific targeted killing effect of this vaccine inhibited melanoma growth and metastasis, leading to a better prognosis [53]. Aikins et al. developed a high-density lipoprotein (sHDL) nanodisc vaccination that included CpG (toll-like receptor 9 agonist), Sox2, Nanog, and ALDH antigenic peptides. This vaccination increases CD8^+^ T cell intratumoral infiltration at the same time lowering the frequency of CSCs and CD4^+^ regulatory T cells in melanoma [54]. This study provides a novel approach for targeting and eliminating CSCs in melanoma. Park et al. suggested a strategy to control paracrine proteins and signaling pathways in adipose-derived stem cells (ADSCs) to attract CD8^+^ CTLs to melanoma, allowing for effective tumor immunotherapy [55]. This work provides insights into the control of stem cell paracrine factors via external stimulation, which may influence the design of stem cell-based cell treatments for cancer.

### Liver Cancer

In hepatocellular carcinoma (HCC), CD8^+^ T cells constitute a majority of TILs [56].

Previous studies have demonstrated that CD8^+^ TIL levels in hepatocellular carcinoma tumor tissue are significantly associated with low recurrence rates and prolonged recurrence-free survival [32, 33]. Tumor endothelial cells (TECs) induce CD8^+^ TIL exhaustion via glycoprotein nonmetastatic melanoma protein B (GPNMB) expression. This suggests that GPNMB in the tumor vasculature may be a novel target for HCC treatment [57]. A recent meta-analysis of 21 observational studies with data from 3509 HCC patients demonstrated that in Asian patients, a high density of CD8^+^ TIL in the tumor causes better overall and disease-free survival (DFS). The lower the level of CD8^+^ TIL infiltration, the larger the tumor size and the later the TNM stage. These results suggest that the CD8^+^ TIL infiltration level can be a reliable indicator for assessing the prognosis of HCC patients [58]. However, there is a need for both comprehensive mechanistic studies and more non-Asian studies to reduce heterogeneity.

A growing body of evidence suggests that CSCs are associated with the origin, growth, metastasis, recurrence, and medication resistance of HCC [59]. High expression of Metadherin (MTDH) increases the invasive and migratory capacity of HCC cells, promoting the growth and self-renewal of CSCs. MTDH also upregulates the expression of PD-L1 via the β-catenin/ lev − 1 signaling pathway and upregulates the transcriptional activity of PD-L1, which in turn inhibits the infiltration of CD8^+^ T cells [60]. MTDH may be a potential molecular marker for HCC. Zhu et al. discovered that HNRNPM knockdown prevents tumorigenesis and reduces cancer stem cell properties of HCC in vitro and in vivo. Furthermore, HNRNPM inhibition substantially improves CD8^+^ T cell activation, whereas HNRNPM-antisense oligonucleotide successfully suppresses WNT/b-catenin, thereby improving anti-programmed cell death protein-1 immunotherapy by promoting CD8^+^ T cell infiltration [61]. Removal of CSCs in HCC and activation of the intrinsic immune response of tumor cells by targeting HNRNPM may be a novel treatment tool of significance.

### Squamous cell carcinoma

Previous studies have extensively demonstrated the prognostic value of CD8^+^ TIL in initial HNSCC [29–31]. A recent study found a significant difference in the level of CD8^+^ TIL infiltration between post-treatment recurrence and initial tumors in HNSCC. The CD8^+^ TIL recurrence to an initial ratio (R/I) can be a good predictor of overall survival (OS) [62]. Recent experimental findings by Mario et al. similarly support that high CD8^+^ TIL infiltration correlates with a better survival outcome [63]. Increased CD8^+^ cell infiltration in invasive oral squamous cell carcinoma is linked to improved patient survival. Additionally, decreased levels of CD8^+^ T cells have been significantly associated with prolonged morbidity, lymph node metastasis, and shorter survival time [64, 65]. Previous studies have reported higher levels of CD8^+^ T-cell infiltration in oral precancerous lesions than that in normal epithelium [66, 67]. Pre-cancerous lesions that progressed to cancer had higher levels of CD8^+^ cells than that of precancerous lesions that did not develop into cancer [68]. Chaves et al. revealed that the evolution of oral precancerous lesions to cancer is linked to a decrease in the infiltration of immune cells, including CD8^+^ T cells, and an upregulation in PD-1 expression in tumor cells [69]. CD8^+^ T lymphocytes are an independent prognostic predictor for oropharyngeal squamous cell carcinoma, and larger levels of infiltrating CD8^+^ T cells are responsible for a better prognosis [70]. PES1 prevents IL-15 expression in esophageal squamous cell carcinoma (ESCC) by interfering with the relationship between ILF3 and IL-15 mRNA, which in turn promotes the breakdown of mRNA. Downregulated IL-15 expression suppresses the infiltration of CD8^+^ CTLs, which in turn promotes ESCC evasion of immune surveillance [71]. Most primary tracheal cancers, such as SCC, possess membrane PD-L1 expression and a high CD8^+^ immune cell infiltration rate. PD-L1 expression is a biomarker for patients with primary tracheal squamous cell carcinomas [72].

Previous findings suggest that CSCs play an important role in chemoresistance, relapse, and metastasis of HNSCC. A recent study identified a long-stranded non-coding RNA (lncRNA) called PVT1, which is highly expressed in CSCs and closely associated with lymph node metastasis in HNSCC. PVT1 inhibits the stimulation of the DNA damage response and induces the recruitment of chemokines by CD8^+^ T cells, as well as prevents CSCs and metastasis by modulating the miR-375/YAP1 axis [73]. By immune checkpoint inhibition, targeting PVT1 may enhance the removal of CSCs, halt metastasis, and impede the progression of HNSCC. In a mouse model of HNSCC, anti-CD276 antibody suppressed tumor development and lymph node metastasis at the same time eliminating CSCs dependent on CD8^+^ T cells. Single-cell RNA sequencing revealed that CD276 blockade alters SCC heterogeneity and lowers epithelial-mesenchymal transition [74]. These results imply that CSCs use CD276 for immunological escape and that targeting CD276 may reduce CSCs in NHSCC. Overcoming cisplatin resistance currently remains one of the key goals of anticancer therapy for many tumors including squamous carcinoma. Targeting MYC using the small molecule inhibitor MYCi975 in cisplatin-treated HNSCC could help eliminate CSCs, prevent metastasis, and overcome cisplatin resistance. Additionally, this tool promotes the infiltration of CD8^+^ T cells within the tumor, further improving the therapeutic efficacy by enhancing anti-tumor immunity [75]. In OSCC, Lequerica-Fernández et al. showed that CD4^+^ and CD8^+^ TILs inhibit NANOG and SOX2 expression, and FOXP3^+^ TILs significantly correlate with Nestin and PDPN expression. For the survival of OSCC patients, the CD8^+^/FOXP3^+^ TILs ratio is an independent prognostic factor [76]. CD8^+^ T cells may act as biomarkers and promising treatment targets for OSCC.

### Breast cancer

Breast cancer is the most frequent type of cancer among women and a heterogeneous entity with numerous biological features associated with prognosis and response to treatment. Previous studies have shown that CD8^+^ TILs possess anti-tumor immune responses in breast cancer [39]. SOX2 has been reported to promote p65 and CCL1 expression in BCSC to recruit Treg in the tumor microenvironment, which in turn may prevent CTL from exerting its effects [77]. Extensive tumor infiltration by CD8^+^ T cells is strongly associated with survival and response to therapy in breast cancer patients [40]. In breast cancer, neurofilament medium (NEFM) transcript expression is downregulated and negatively correlates with its DNA methylation. NEFM transcript expression correlates with an increase in CD8^+^ T cells. NEFM methylation contributes to the poor prognosis of breast cancer by attenuating the infiltration of immune cells, including CD8^+^ T cells [78]. In patients with HER2-negative breast cancer who express HLA class 1 on their tumors, baseline tumor infiltration by CD8^+^ T lymphocytes is associated with improved DFS and greater pCR rates after neoadjuvant treatment [79]. Furthermore, in triple-negative breast cancer (TNBC), high mesenchymal CD8^+^ TIL levels are favorable prognostic factors for RFS and overall survival (OS) [80].

Previous studies revealed that BCSCs can influence the tumor microenvironment and the outcomes of immunotherapy by interacting with immune cells that infiltrate tumors, including CD8^+^ T cells [81]. CHI3L1, which is generated from triple-negative breast cancer stem cells (TN-BCSCs), causes immunosuppression by improving CTLA4 expression in T cells via MAF and decreasing CD8^+^ T cell cytotoxicity. Furthermore, CTLA4^+^ T cells may produce S100A4, promoting the stemness of TNBC cells [14] Targeting CHI3L1 to restore the tumor-killing activity of CD8^+^ T cells might be a viable approach for treating TNBC. A dendritic cell vaccine transfected with CD133 mRNA was shown to attenuate the stemness of CSCs by upregulating the expression of CD8^+^ TILs in TNBC in mice, in turn inhibiting tumor growth and causing longer survival time [82]. This study may add to the therapeutic options for TNBC. Interleukin 20 receptor subunit alpha (IL20RA) increases the ratio of SP to ALDHbr in breast cancer cells, improves sphere-forming capacity, stimulates the production of key stem cell genes such as Sox2 and Oct4, and boosts chemoresistance. Additionally, IL20RA caused the signaling pathway of Janus kinase 1 (JAK1)-STAT3-SOX2, upregulating PD-L1 expression and decreasing the recruitment of immune cells, including CD8^+^ T cells [83]. Targeting IL20RA to improve breast cancer treatment may be a potential novel strategy. An evolutionarily conserved RNA-binding protein called Lin28B, which is extensively expressed in embryonic stem cells, increases the stemness, migration, and invasion of breast cancerous tumors [84]. Recent findings suggest that Lin28B can suppress the proliferation, infiltration, and activation of CD8^+^ T cells by causing neutrophil infiltration and N2 transformation, thereby creating an immunosuppressive microenvironment [85]. This result adds to the evidence supporting the role of Lin28B in the initiation of breast cancer metastasis. DCLK1 upregulates IL-6 expression and STAT3 activation in TNBC cells, which promotes CSC stemness and inhibits CD8^+^ T cell activity. Inhibition of the IL-6/STAT3 pathway by IL-6R antagonists, Tocilizumab, or STAT3 inhibitors, S31-201 abrogates the DCLK1-promoted malignant phenotype of TNBC cells [86].

### Lung cancer

As one of the most common malignant tumors, lung cancer frequently affects the alveolar and bronchial mucosal epithelium. Previous studies have shown a positive correlation between increased levels of CD8^+^ T-cell infiltration and a better prognosis for lung cancer [37]. Abnormal expression of β-catenin protein is significantly increased in NSCLC is linked to a poor prognosis. The presence of β-catenin protein in non-small cell lung cancer (NSCLC) also prevents CD8^+^ T cell and neutrophil invasion, contributing to the tumor immune microenvironment [87]. Ye et al. discovered that high infiltrating levels of CD8^+^ T lymphocytes signify a favorable outcome for individuals with lung adenocarcinoma (LUAD) [38]. CELSR3 is an important signaling molecule in the WNT/PCP pathway hypothesized to participate in tumorigenesis and metastasis. In previous research, DC-derived CCL17 was found to increase the contact between DCs and CD8^+^ T cells, therefore activating CD8^+^ T cells [88, 89]. Li et al. found that down-regulation of CELSR3 significantly inhibits the proliferation, migration, and invasive capacity of LUAD cells. On the other hand, its down-regulation might improve LUAD cell proliferation, metastasis, and invasion by up-regulating the CCL17 /CCR4 axis to increase the level of CD8^+^ T cell infiltration in LUAD [90]. More experiments are necessary to validate the diagnostic and immunotherapeutic value of CELSR3 in LUAD.

According to Corgnac et al., NSCLC reduces the cell surface of the CD103 integrin ligand E-cadherin, starting an epithelial-to-mesenchymal transition program that enabled CSC to withstand particular CD8^+^ CD103^+^ TRM cell-mediated cytotoxicity and maybe also resistance to cancer immunotherapy [91]. Targeting CSC EMT combined with immune checkpoint blockade is anticipated to be a future immunotherapy for NSCLC. In small-cell lung cancer (SCLC), chemotherapy-resistant CSCs are also a major cause of drug resistance and aggressiveness [92]. Mesenchymal CSC-like SCLC cells stimulate CTLs immunologically, which causes an increase of co-inhibitory receptors on CTLs and T-cell fatigue. CSC-like SCLC cells upregulate PD-L1 and PD-L2 expression, limiting CTL responses, in response to CTL activation and IFN-γ production [93]. A deeper understanding of the immunoregulatory mechanism regulated by CSC-like cells may result in new cancer immunotherapy methods for SCLC patients.

### Colorectal Cancer

Colorectal cancer (CRC) is one of the most prevalent malignancies of the digestive tract. For colorectal cancer, an immunoscore developed by integrating T-cell data from the center of the tumor with the margins of infiltration has confirmed a potent biomarker for assessing survival risk [94]. In CRC, the presence of CD8^+^ T cells as an independent prognostic factor is linked to improved overall and cancer-specific survival and may be adopted in the treatment of CRC [34–36, 95]. CCL5 deficiency inhibits colorectal cancer growth and metastasis by promoting the infiltration of CD8^+^ T cells into the central region of the tumor [96]. CCL5 knockdown could help in anti-colorectal cancer treatments. Through immunohistochemistry, and investigation of 155 colorectal cancer tissues, Xue et al. found that CD8^+^ T-cell infiltration is considerably decreased in tumors with high expression of β-catenin [97]. This implies that β-catenin signaling may mediate resistance to immunotherapy in colorectal cancer and that targeting β-catenin combined with PD -1 immunotherapy for colorectal cancer is a promising direction. Noh et al. found that in colorectal adenocarcinomas, high levels of CD8^+^ TILs similarly imply a better prognosis [98]. Generally, the total infiltration level of CD8^+^ TILs and differences in infiltration at various tumor sites may predict prognosis for patients with colorectal cancer. Nevertheless, additional prospective studies are necessary.

In TAMs, an inhibitor of differentiation 1 (ID1) prevents CD8^+^ T cell infiltration and STAT1-mediated transcription of SerpinB2 and CCL4, hence preserving tumor stemness [99]. Reducing ID1 expression improves colorectal cancer progression and improves tumor sensitivity to immunotherapy and chemotherapy. Mennonna et al. discovered that the SMAD4^V370A^ somatic mutation in colorectal cancer produces a naturally processed novel epitope that can be detected by differentiated and CSC-cultured autologous CD8^+^ T cells [100]. This study provides a new approach for quantitatively identifying novel epitopes for CRC mutations. On the other hand, Miyamoto et al., identified the ASB4 antigen, a gene for which is expressed in colorectal cancer CSCs but not in cells that differentiate into non-CSCs. The peptide epitope of ASB4, which is not expressed in healthy tissues, causes a CTL response that destroys colorectal cancer CSCs while preserving non-CSCs [101]. Immunotherapy based on CTLs may help in preventing colorectal cancer recurrence.

In summary, tumor-infiltrating CD8^+^ T cells play a role in the development of various cancers and may act as a prognostic indicator. Comprehensive research on the mechanisms of CD8^+^ T cells in these cancers and the development of novel CD8^+^ T cell-based immunotherapies may improve the understanding of these cancers and their treatments. The involvement of CSC in these processes is equally important and worth exploring.

## Molecular mechanisms by which tumor stem cell (CSC) labeling regulates CD8^+^ T cell activity

Tumor stem cell (CSC) markers are not only used to identify CSCs, but also shape the immunosuppressive tumor microenvironment (TME) by directly or indirectly modulating the function of CD8^+^ T cells through multiple mechanisms. The following is a detailed description of its mechanism of action (Table 1).

**Table 1:** Summary table of core mechanisms of CSC regulation of CD8^+^ T cell activity

Summary Table of Core Mechanisms of CSC Regulation of CD8 ^+^ T Cell Activity				
Classification of mechanisms		Key molecules/pathways	Effect on CD8^+^ T cells	experimental evidence
Direct signal suppression	PD-L1/PD-1 pathway	PD-L1 (CSC), PD-1 (T cells)	Inhibits TCR signaling, reduces granzyme B/perforin secretion, and promotes depletion phenotype (TIM-3/LAG-3↑)	Immune checkpoint inhibitors targeting PD-1/PD-L1 have demonstrated efficacy in the treatment of a variety of tumors, including HCC [148].
	CD44/hyaluronic acid (HA) interactions	CD44(CSC)、HA (TME)	Inhibits T cell migration (RhoA/ROCK pathway activation), down-regulates the co-stimulatory receptor CD28, and impairs TCR signaling	Inhibition of OPN (one of the ligands for CD44) promotes CD8^+^ T cell activation [149]
	CD133/STAT3 pathway	CD133(CSC), STAT3 (T cells)	Activation of STAT3 signaling induces IL-10 secretion from T cells, forming an autocrine inhibitory loop	CAR-T therapy targeting CD133-positive CSC has been shown to be beneficial in the treatment of many tumors, including HCC [150]
Secretory factor inhibition	TGF-β/Smad pathway	TGF-β (CSC), TβR (T cells)	Downregulation of T-bet transcription factors and reduction of IFN-γ secretion; induction of T-cell depletion phenotype (PD-1 + TIM-3+)	TGF-β inhibits several aspects of T cell proliferation, activation and effector functions [151]
	IL-10/STAT3 pathway	IL-10 (CSC), IL-10R (T cells)	Inhibits mTOR activity, induces metabolic reprogramming (oxidative phosphorylation ↑, glycolysis ↓), impairs effector functions	Inhibition of IL-10 Enhances CAR-T Efficacy [152]
	Exosome-mediated inhibition	miR-21, FasL (CSC exosome)	miR-21 targets PTEN and activates the PI3K/AKT pathway to promote T cell depletion; FasL induces T cell apoptosis	circTRPS1-derived exosomes knock down BCa cells, prevent CD8^+^ T-cell depletion, and suppress the malignant phenotype of BCa cells [153]
metabolic interference	Tryptophan depletion (IDO/Kyn)	IDO1 (CSC), kynurenine (Kyn)	Activation of GCN2 kinase (integrative stress response) and inhibition of mTORC1 activity → T cell proliferation arrest; activation of AhR induces Treg differentiation	Abrine acts as an IDO1 inhibitor with inhibitory effects on immune escape and synergizes with anti-PD-1 antibodies in the treatment of HCC [154].
	lactic acid accumulation	Lactate (CSC), MCT (T cells)	Inhibition of mTOR signaling and HIF-1α stability → IFN-γ secretion ↓; acidified microenvironment inhibits T cell infiltration	Co-administration of MCT4 inhibitor and ICB enhances immune cell infiltration, T-cell function [155]
	Lipid metabolism interference	FFA、PGE2(CSC)	FFA inhibits glycolysis and mitochondrial respiration via PPARγ; PGE2 inhibits TCR signaling	COX-2 inhibitor enhances T-cell function [156]
Immunosuppressive cell recruitment	CCL2/CCL5-CCR2/CCR5	CCL2/CCL5(CSC)	Recruitment of Treg and MDSCs → Treg inhibits T cells via CTLA-4; MDSCs secrete Arg1/iNOS to deplete arginine and inhibit TCR signaling	ETV4 promotes CCL2 expression and can increase MDSCs infiltration [128]
	M2-type macrophage polarization	IL-4、IL-13(CSC)	Induction of IL-10 and PD-L1 secretion by macrophages and inhibition of CD8^+^ T cell activity	CSF1R inhibitors block macrophage polarization [157]
epigenetic regulation	DNA methylation	DNMT (CSC induction)	IFN-γ and TNF-α gene promoter hypermethylation → effector gene silencing	DNMT1 deletion protects mice from mammary tumorigenesis by limiting the CSC pool [158]
	histone modification	HDAC (CSC exosome carrying)	Histone deacetylation → chromatin tightening, inhibition of granzyme B (GZMB) and perforin (PRF1) gene expression	HDAC inhibitor (Vorinostat) enhances CAR-T efficacy [135]

### CSC directly regulates CD8 + T cell activity

#### CSC surface markers and immune checkpoint signaling

PD-L1 expressed on the surface of tumor cells interacts with PD-1 expressed on the surface of T cells which further inhibits multiple signaling pathways [102]. CSC upregulates PD-L1 expression by activating signaling pathways such as STAT3, NF-κB or HIF-1α. For example, CD44 binding to hyaluronic acid activated the PI3K/AKT/mTOR pathway and induced PD-L1 expression. PD-L1 binding to PD-1 on the surface of CD8^+^ T cells activates SHP-2 phosphatase and inhibits TCR signaling pathways (e.g., ZAP70, PI3K/AKT phosphorylation) [103], leads to decreased T cell proliferation, decreased secretion of cytotoxic molecules (granzyme B, perforin), and promotes the expression of T cell depletion markers (e.g., TIM-3, LAG-3) [104, 105].

Studies have also demonstrated that some CSC also affect CD8^+^ T cell activity by influencing a number of other immune checkpoint molecules and thus: expression of CD155 (PVR) by CSC, which binds to the TIGIT of CD8^+^ T cells and inhibits their activation and IFN-γ secretion [106, 107]. In addition, CSC secrete Galectin-9, which binds to the TIM-3 receptor and induces apoptosis in CD8^+^ T cells [104]. 

#### CSC labeling-mediated Inhibition of co-stimulatory signals

Binding of CD44 on the surface of CSC to hyaluronan HA in TME activates the RhoA/ROCK pathway and inhibits migration and infiltration of CD8^+^ T cells [108]. Meanwhile, the CD44-HA complex attenuates TCR signaling by down-regulating CD28 (co-stimulatory receptor) expression.CD133 activates STAT3 via Src kinase and induces CD8^+^ T cells to express IL-10 and TGF-β, forming an autocrine inhibitory loop that suppresses autoactivation and cytotoxicity [109].

### CSC secretion of immunosuppressive factors regulates T cell function

#### Cytokine-mediated immunosuppression

CSC secretes TGF-β, which activates the Smad2/3 pathway in CD8^+^ T cells via the TGF-β receptor (TβR), inhibits the expression of T-bet (a key transcription factor for Th1 differentiation), and reduces IFN-γ and granzyme B production [110]. TGF-β also induces differentiation of CD8^ +^ T cells towards a depleted phenotype (PD-1 + TIM-3+) [111].

In addition, CSC secrete IL-10 and IL-35, which activate IL-10R and IL-35R on the surface of CD8^+^ T cells, and inhibit mTOR activity through the STAT1/STAT3 pathway, leading to metabolic reprogramming (e.g., enhanced oxidative phosphorylation, inhibition of glycolysis) and weakening effector functions [103].

#### Exosomes transmit inhibitory signals

Exosomes released by CSC carry PD-L1, non-coding RNAs (e.g., miR-21, miR-214) and immunosuppressive proteins (e.g., FasL). miR-21 targets PTEN, which activates the PI3K/AKT pathway, and the increased expression of PD-L1 on the cell surface leads to decreased T-cell proliferation and increased apoptosis, promoting T-cell depletion [112]. FasL binds to the Fas receptor of CD8^+^ T cells and induces apoptosis [113].

### Metabolic reprogramming inhibits CD8^+^ T cell function

#### Nutrient competition and metabolic repression

CSC highly expresses indoleamine 2,3-dioxygenase (IDO), which catalyzes the catabolism of tryptophan to kynurenine [114]. Tryptophan depletion activates GCN2 kinase in CD8^+^ T cells, triggering the integrative stress response (ISR), which inhibits mTORC1 activity and leads to T cell proliferation arrest [115, 116]. Kynurenine induces CD8^+^ T cell apoptosis and promotes regulatory T cell (Treg) differentiation via the aryl hydrocarbon receptor (AhR) [117]. In addition, CSC secretes large amounts of lactic acid through the Warburg effect, acidifying the TME [118]. Lactate enters CD8^+^ T cells via MCT1, inhibits mTOR signaling and HIF-1α stability, reduces IFN-γ secretion, and induces T cell functional exhaustion. In the highly glycolytic tumor microenvironment, lactate promoted PD-1 expression in regulatory T cells and further inhibited CD8^+^ T cell activity [119].

#### Disruption of lipid metabolism

CSC release free fatty acids (FFA) and oxidized lipids (e.g., prostaglandin E2, PGE2), which inhibit glycolysis and mitochondrial respiration and impair energy metabolism in CD8^+^ T cells via the PPARγ pathway [103, 120].

### Recruitment and activation of immunosuppressive cells by CSC

**Fig. 3:**
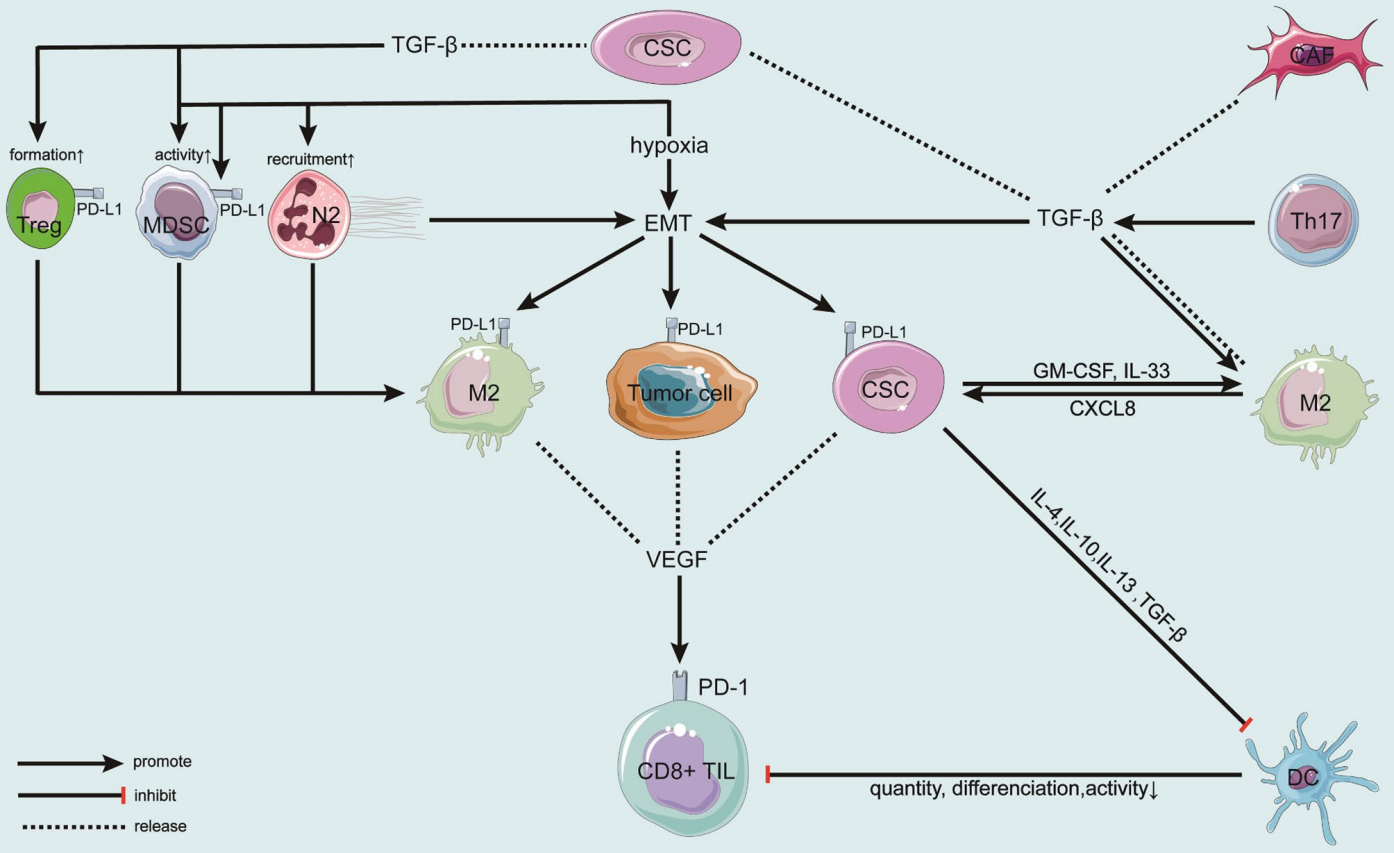
Interactions between cancer stem cells (CSCs) and immune cells in the tumor immune microenvironment (TIME) CSCs enhance the activity of type 2 macrophages (M2), type 2 neutrophils (N2), and myeloid-derived suppressor cells (MDSCs) by producing the transforming growth factor-β (TGF-β), thereby promoting the epithelial-mesenchymal transition (EMT) of tumor cells. EMT is positively correlated to the expression of programmed death ligand 1 (PD-L1) on M2 macrophages and tumor cells, which damages the function of CD8^+^ T cells by increasing the activity of PD-1 and stimulating the release of vascular endothelial growth factor (VEGF). Moreover, CSCs generate immunosuppressive factors such as IL-4, IL-10, and IL-13 to prevent the maturation of dendritic cells (DCs), which weakens the activation of CD8^+^ TILs

Growing evidence suggests a role for CSC in the immune microenvironment (Fig. 3). Tumor-associated macrophages (TAM) and myeloid-derived suppressor cells (MDSC) often stimulate cancer growth and evade the immune system by suppressing effector cells. CD8^+^ regulatory T cells (CD8^+^ Tregs), which have important immunosuppressive functions, are effective in blocking overreactive immune responses and maintaining the body’s immune homeostasis [121].

#### Raising Tregs and MDSCs

CSCs secrete CCL2 and CCL5 and recruit Treg and myeloid-derived suppressor cells (MDSCs) via CCR2/CCR5 receptors.T he presence of CSCs and Tregs positively correlates with malignancy, indicating that these cell types may interact to promote an immunosuppressive environment [122, 123]. In glioblastoma, CSCs mediate Treg cell infiltration through co-stimulatory molecules PD-L1, soluble galactin-3, and TGF-β secretion [124]. Furthermore, by modifying the TME-mediated synthesis of cytokines such as IL-6, IL-8, and the chemokine CCL5, CSCs influence the Th17/Treg balance [125].

CSCs increase G-CSF in a synergistic breast tumor model, which is responsible for luring MDSCs to the tumor site [126]. In addition, MDSCs prevent T cell activation and provide breast cancer cells with stem-like characteristics using NO to stimulate the IL-6/STAT3 and NO/NOTCH signaling pathways [127]. MDSCs impede T-cell growth and activation and contribute to T-cell dysfunction. By upregulating the expression of immunological checkpoint molecules such as PD-L1, CTLA-4, VISTA, Gal-9, and CD155, MDSCs impede tumor-infiltrating CD8^+^ T-cell-mediated anti-tumor immunity. Xia et al. discovered that in hepatocellular carcinoma, ETV4 upregulation promotes PD-L1 and CCL2 expression, increases infiltration of MDSCs, and inhibits CD8^+^ T cell aggregation [128].

#### M2-type macrophage polarization

CSCs secrete macrophage chemo-attractants, which are important for TAM recruitment and M2 polarization [129]. M1-polarized macrophages produce pro-inflammatory cytokines, which increase inflammation, whereas M2-polarized macrophages promote tumor development and metastasis [130]. TAMs are functionally similar to M2-polarized macrophages. They secrete limiting chemokines such as TGF-β, prostaglandins, IL-10, or reactive oxygen species (ROS) to suppress CD8^+^ T cell activation and proliferation [131, 132].

### Epigenetic regulation and T cell depletion

DNA methylation is one of the most characteristic epigenetic modifications regulating gene transcription [133]. Methylation of many genes is associated with normal cell development and differentiation [134]. SC-secreted TGF-β induces DNA methyltransferase (DNMT) expression in CD8^+^ T cells, leading to hypermethylation of the promoters of IFN-γ and TNF-α genes and silencing of their expression. HDACs (histone deacetylases) carried by CSC exosomes enter CD8^+^ T cells and reduce chromatin accessibility of effector genes (e.g. GZMB, PRF1). Pathania R et al. demonstrated in a mouse model of breast cancer that the combination of the DNMT inhibitor 5-azacytidine and the HDAC inhibitor butyrate significantly reduced CSC abundance and increased overall survival in this mouse model [135].

Overall, the above mechanisms provide a theoretical basis for the development of immune-combination strategies targeting CSC. Potential future research directions may include targeting CSC markers with dual blockade of immune checkpoints (e.g., anti-CD44 + anti-PD-1). The development of metabolic modulators (e.g., IDO inhibitors, lactate dehydrogenase inhibitors) in combination with immunotherapy would be equally helpful. There is also value in utilizing single-cell sequencing to resolve the dynamic interplay network of CSC and CD8^+^ T cells.

## CD8^+^ T cells, cancer stem cells and immunotherapy

**Fig. 4:**
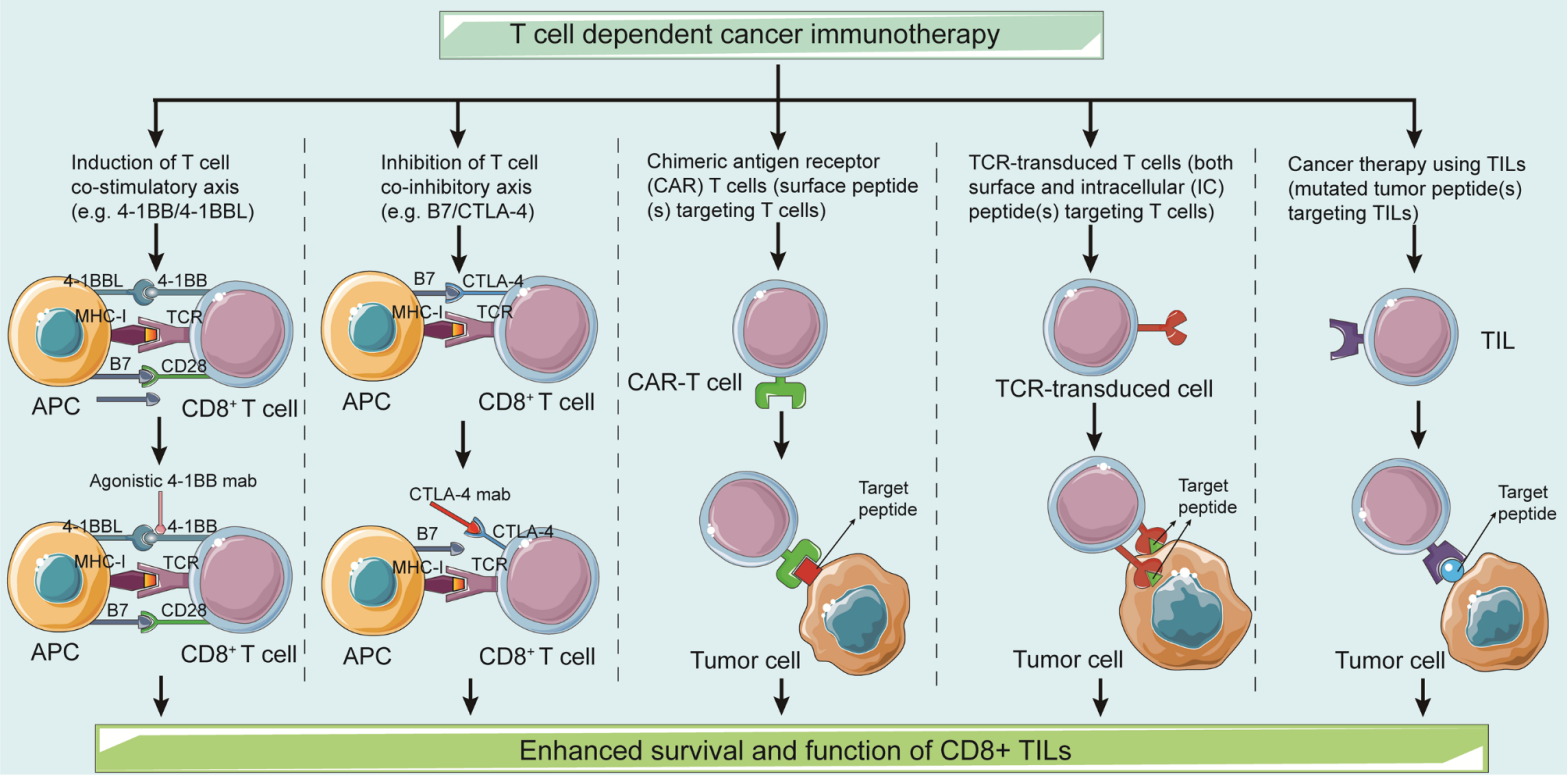
T cell dependent cancer immunotherapy Effective strategies to enhance CD8^+^ T cell infiltration and effector cell function include autologous back-treatment with chimeric antigen receptor (CAR) T cells, TCR-transduced T cells, and tumor-infiltrating T cells (TILs). Inhibiting antibodies can also be used to suppress the T cell co-inhibitory axis (e.g., B7/CTLA-4). Antagonistic antibodies can induce T cell co-stimulatory axis (e.g., 4-1BB/4-1BBL)

CD8^+^ T cells are the primary immune cells that regulate immune surveillance and antigens identification in cancer cells and malignant tumors. Following TCR antigen recognition, CD8^+^ T cells are activated, rapidly proliferate and differentiate into cytotoxic T lymphocytes (CTLs), causing damage to cancer cells via cell-cell contact. CD8^+^ T cell malfunction is more prevalent in solid malignancies. Researchers have focused on identifying immunotherapeutic strategies that can both boost or restore the immunological activity of CD8^+^ T cells and expand their penetration. Several techniques have been proposed, including the use of agonistic antibodies, T-cell co-stimulatory chemicals, chimeric antigen receptor (CAR) T cells, TCR-transduced T cells, checkpoint inhibitor (CPI) antibodies, and TIL-based cancer therapies (Fig. 4). This section has been discussed in some detail in past studies.

CSC is often resistant to conventional therapies and can lead to recurrence and metastasis, while CD8 T cells are key to the immune system’s ability to attack tumors. Therapies combining the two may include directly targeting the antigens of CSC to aid CD8 T-cell recognition, or unlocking the immunosuppressive environment induced by CSC to make CD8 T-cells more effective.

### Core strategies for combining CD8^+^ T cells with CSC targeting

#### Targeting CSC-specific antigens to activate CD8^+^ T cells

Designing immunotherapy through CSC surface-specific markers (e.g., CD133, CD44, EpCAM, etc.) allows CD8 + T cells to directly recognize and clear CSCs. The main component of CAR-T is the chimeric antigen receptor (CAR), which endows T cells with the capacity to identify tumor antigens in an HLA-independent manner, allowing them to recognize several target antigens compared with the native T-cell surface receptor (TCR) [136]. In contrast to TCR-T, CAR-T is more commonly prescribed in patients with some HLA types and cancer types that lack HLA expression, which is a common strategy of cancer immune escape. Basic CARs include tumor-associated antigen (TAA)-binding domains (usually scFv fragments derived from the antigen-binding region of monoclonal antibodies), extracellular hinge domains, transmembrane domains, and intracellular signaling domains [137]. In some cases, CAR-T may cause severe adverse effects, such as the cytokine release syndrome and neurotoxicity. CAR-T can kill cancer cells by recognizing and binding to TAA, implying that it is an ideal candidate cell for developing immunotherapy targeting CSC. For studies investigating, CAR-T cells effectively eliminated CSC, but not normal stem cells or showed low cytotoxicity to them [138–141]. Several CSC markers for CAR-T cell therapy have been reported in preclinical studies including CD133, CD166, CD20, CD38, CLL-1, EpCAM, CD123, CD171, ROR1, CD44, CD47, CD117 and c-Met [142]. Zhao et al. The STAT3 inhibitor nikrolamide (Ni) and an experimental iron metastatic drug (1 S, 3R)-RSL3 (RSL3) were incorporated via biomineralization into hyaluronic acid-modified amorphous calcium phosphate (ACP) nanocells (CaP-PEG-HA@Ni/RSL3), which can be over-expressed by CD44 CSCs recognized and released in a synchronized manner. Nickel inhibited the endogenous STAT3-PD-L1 axis of CSCs, thereby stimulating adaptive immunity, and enhanced the secretion of gamma interferon (IFNγ) by CD8^+^ T-cells, which resulted in the down-regulation of SLC7A11 and SLC3A2 and impeded the biosynthesis of glutathione. This study provides a cooperative ferroptosis-immunotherapy approach for the treatment of refractory cancers [143].

#### Release of CSC-mediated immunosuppression

Blocking the inhibitory effects of CSC on CD8^+^ T cells via PD-L1, cytokines (IL-10, TGF-β), or metabolites (e.g., lactate) would help promote CD8^+^ T cell activity. The use of immune checkpoint inhibitors (e.g., anti-PD-1/PD-L1 antibodies) to restore CD8 + T cell activity as well as targeting inhibitory factors secreted by CSC (e.g., TGF-β inhibitors) may be helpful in tumor therapy.

#### Combination therapies to enhance synergies

A combined strategy of CSC-targeted therapy and T-cell immune activation simultaneously removes CSCs and enhances the immune response.IDO inhibitors block tryptophan metabolism inhibition and restore T-cell function, while combining with PD-1 antibody to enhance T-cell activity.TGF-β inhibitors block CSC-mediated T-cell depletion, while CSC vaccines are used to induce specific immune responses. Lysovirus lyses tumor cells to release antigens while CSC vaccine delivers CSC-specific antigens that activate DC cells and promote CD8^+^ T cell cross-presentation. This approach induces long-lasting immune memory and prevents recurrence.

### Progress of clinical trials

Clinical trials provide a quantitative assessment of the safety and efficacy of medicines and offer patients safer and more effective treatment options. They play an important role in the practice of modern medicine. Through clinical trials, medical researchers can collect data, evaluate the effectiveness of treatments, and provide better healthcare for patients [144, 145]. This article summarizes some of the relevant clinical trials. (Table 2)

**Table 2:** Clinical trials combining CD8^+^ T cells with CSC targeting

ID	Institute	Title	Antigen	Type of cancer	Phase	Enrollment	Publications(PMID)	Status
NCT02541370	Chinese PLA General Hospital	Treatment of Relapsed and/or Chemotherapy Refractory Advanced Malignancies by CART133	CD133	BCA、OC、CRC、HCC、PAAD、AL Malignant Brain Neoplasm	Phase 1/2	20	[146]	Completed
NCT03013712	First Affiliated Hospital of Chengdu Medical College	A Clinical Research of CAR-T Cells Targeting EpCAM Positive Cancer	EpCAM	CRC、ESCA、PAAD、PRAD、GC、HCC	Phase 1/2	60		Unknown status
NCT04650451	Bellicum Pharmaceuticals	Safety and Activity Study of HER2-Targeted Dual Switch CAR-T Cells (BPX-603) in Subjects With HER2-Positive Solid Tumors	HER2	GC、BC	Phase 1/2	220		Recruiting
NCT03450044	Fundación Salud de los Andes	Immunogenicity and Safety of DCs in Breast Cancer		BC	Phase 1/2	15	[159]	Completed
NCT04060342	Gossamer Bio, Inc	GB1275 Monotherapy and in Combination With an Anti-PD1 Antibody in Patients With Specified Advanced Solid Tumors or in Combination With Standard of Care in Patients With Metastatic Pancreatic Adenocarcinoma	CD11b、PD-1	PAAD	Phase 1	61	[147]	Terminated
NCT03348904	Incyte Corporation	Nivolumab and Epacadostat With Platinum Doublet Chemotherapy Versus Platinum Doublet Chemotherapy in Non-Small Cell Lung Cancer	PD-1、IDO1	NSCLC	Phase 3	2		Terminated
NCT03875313	Calithera Biosciences, Inc	Study of CB-839 (Telaglenastat) in Combination With Talazoparib in Patients With Solid Tumors	Glutaminase、PARP	RCC、TNBC、CRC、ccRCC	Phase 1/2	33		Terminated

#### CSC-targeted CAR-T cell therapy

CD133 is a classical marker of CSC, which is highly expressed in many solid tumors such as glioblastoma, hepatocellular carcinoma, and pancreatic cancer, but lowly expressed in normal tissues (e.g., hematopoietic stem cells, endothelial progenitor cells.) The clinical trial (NCT02541370) conducted by Dai H, et al. focuses on the treatment of advanced solid tumors (hepatocellular carcinoma, pancreatic carcinoma, etc.) with CD133 CAR-T cells. The trial had a good initial safety profile and tumor markers decreased in some patients, but efficacy was limited by target heterogeneity [146]. EpCAM is highly expressed in CSCs of epithelial origin tumors such as gastrointestinal tract cancer and breast cancer, but it is widely present in normal epithelial cells and requires careful selection of indications. Another related clinical trial (NCT03013712) focused on EpCAM CAR-T for the treatment of EpCAM-positive solid tumors (gastric cancer, colorectal cancer). Disease stabilization was achieved in some patients, but target selection needs to be optimized to reduce normal tissue toxicity.HER2 is overexpressed in breast cancer, gastric cancer CSC. A clinical study (NCT04650451) investigated the safety, tolerability, and clinical activity of HER-2-specific dual-switch CAR-T cells, BPX-603, which were used with rimiducid in previously treated patients with locally advanced or metastatic solid tumors with HER-2 amplification/overexpression. Another clinical trial targeting HER2 focused on the safety and tolerability of CCT303-406 CAR-modified autologous T cells (CCT303-406) in subjects with relapsed or refractory stage IV metastatic HER2-positive solid tumors.

#### CSC vaccines combined with immune checkpoint inhibitors

The role of CSC vaccines is mainly to deliver CSC-specific antigens (e.g., CD133, ALDH1A1, SOX2), to doubt dendritic cells (DCs), to promote antigen cross-presentation to CD8^+^ T cells, and to induce CSC-specific memory T cells, which reduces the risk of recurrence.The advantage of CSC vaccines in combination with immune checkpoint inhibitors is that ICIs deregulate T cell suppression (e.g., anti-PD-1/PD-L1 antibodies block the PD-1/PD-L1 pathway and reverse vaccine-induced T cell depletion). In addition, ICI can amplify the antigenic response by enhancing vaccine-induced T-cell clonal expansion and tumor infiltration.

#### Bispecific antibody (BsAb) targeting CSCs and T cells

Bispecific antibody (BsAb) is a special antibody that can bind two different antigens at the same time, and has a mechanism of action that mediates immune cell killing, dual-targeted signaling blockade, and promotes the formation of functional complexes of proteins, etc. BsAb usually contains two antigen-binding domains: the antigen-binding domain that targets CSCs: recognizes specific markers on the surface of CSCs (e.g., EpCAM, CD133, CD44). Antigen-binding domain that activates T cells: binds CD3 molecules on the surface of T cells and triggers the TCR signaling pathway. EpCAM/CD3 dual antibody was used in the trial (NCT03450044) to treat EpCAM-positive solid tumors.

#### Targeting CSC metabolism and the immune microenvironment

In the trial (NCT04060342), researchers treated solid tumors with GB1275, a CD11b activator, in combination with anti-PD-1. GB1275 mediates the migration of MDSCs to the TME through activation of CD11b, which in turn affects resistance to ICI and other anticancer therapies [147]. Blocking IDO1 Activity Restores Tryptophan Levels and Reduces Kynurenine, Reversing T Cell Suppression. A clinical trial (NCT03348904) focused on evaluating the efficacy and safety of the combination of nivolumab plus epacadostat in combination with platinum chemotherapy compared with platinum chemotherapy alone, in participants with treatment-naïve Stage 4 or recurrent non-small cell lung cancer (NSCLC). Inhibition of glutaminase (GLS) blocks CSC energy supply and reduces glutamate secretion. A clinical trial (NCT03875313) investigated the recommended phase 2 dose (RP2D), safety and tolerability, pharmacokinetics (PK), and clinical activity of the glutaminase inhibitor CB839 (Telaglenastat) versus the poly(adenosine diphosphate ribose polymerase) (PARP) inhibitor talazoparib in participants with advanced/metastatic solid tumors. MCT1 inhibitors reverse tumor microenvironment acidification by blocking lactate efflux. A clinical trial (NCT01791595) using AZD3965 monotherapy in advanced solid tumors showed that it was well tolerated, with a decrease in serum lactate levels in some patients, but that single-agent efficacy was limited, and combination immunotherapy was needed.

### Challenges and future directions

Many challenges remain in developing new therapies for tumor treatment by targeting CSC. CSC may express different markers in the same tumor, requiring the development of multi-targeted therapies (e.g., dual CAR-T or combination vaccines.) CSC can evade immune recognition through phenotypic switching (e.g., epithelial-mesenchymal transition) or down-regulation of target antigens to achieve immune escape, and CSC markers (e.g., CD133, EpCAM) are under-expressed in normal stem cells, which requires improved targeting specificity. Possibly effective measures we can do in the future are adjusting the timing and dosage such as sequential immune checkpoint inhibitors after CAR-T cell therapy, exploring CSC-specific neoantigens or metabolic pathways (e.g., Wnt/β-catenin). Designing neoantigenic vaccines based on the mutational profile of a patient’s CSC may also be helpful. Enhancing the efficiency of CD8^+^ T cell initiation by nanoparticle delivery of CSC antigens and immune adjuvants to lymph nodes is also a promising research direction.

## Conclusion

Overall, this study demonstrates that tumor-infiltrating CD8^+^ T cells exert tumor-suppressive effects in many cancers. CD8^+^ TILs have been shown to participate in the development of several cancers, and high levels of CD8^+^ TILs are associated with better prognosis in most tumors. The antitumor activity of CD8^+^ T cells in the TME is driven by processes such as antigen presentation, effective T cell start, transit, differentiation, and function. However, the immunosuppressive environment established by several TME factors suppresses the killing effect of CD8^+^ TILs on tumor cells, which aggravates tumor progression. Several types of cancer immunotherapy have been shown to successfully restore and enhance antitumor immunity in CD8^+^ T cells. Given the TME’s immunosuppressive effects, many of these therapies are ineffective or have limited efficacy. CSCs play an important role in the immunosuppressive complex seen in the tumor microenvironment. The presence of cancer stem cells in the TME decreases the antitumor activity of the immune system, specifically CD8^+^ TILs. Although several studies have explored the behavior of CSC, specific CSCs-based treatments have not been effectively investigated. Combination therapies designed to enhance effector CD8^+^ T cell activity while decreasing TME’s immunosuppressive effects are increasing being developed. Meanwhile, additional research is needed to determine the role of CSCs in immunomodulation of cancer patients and create novel treatment methods.

## Data Availability

Not applicable.

## References

[1] Wherry EJ, Ha S-J, Kaech SM, Haining WN, Sarkar S, Kalia V, et al. Molecular signature of CD8 + T cell exhaustion during chronic viral infection. Immunity. 2007;27:670–84. 17950003 10.1016/j.immuni.2007.09.006

[2] Barber DL, Wherry EJ, Masopust D, Zhu B, Allison JP, Sharpe AH, et al. Restoring function in exhausted CD8 T cells during chronic viral infection. Nature. 2006;439:682–7. 16382236 10.1038/nature04444

[3] Lapidot T, Sirard C, Vormoor J, Murdoch B, Hoang T, Caceres-Cortes J, et al. A cell initiating human acute myeloid leukaemia after transplantation into SCID mice. Nature. 1994;367:645–8. 7509044 10.1038/367645a0

[4] Bonnet D, Dick JE. Human acute myeloid leukemia is organized as a hierarchy that originates from a primitive hematopoietic cell. Nat Med. 1997;3:730–7. 9212098 10.1038/nm0797-730

[5] Prager BC, Xie Q, Bao S, Rich JN. Cancer stem cells: the architects of the tumor ecosystem. Cell Stem Cell. 2019;24:41–53. 30609398 10.1016/j.stem.2018.12.009 PMC6350931

[6] Plaks V, Kong N, Werb Z. The cancer stem cell niche: how essential is the niche in regulating stemness of tumor cells? Cell Stem Cell. 2015;16:225–38. 25748930 10.1016/j.stem.2015.02.015 PMC4355577

[7] Alba J, D’Abramo M. The full model of the pMHC-TCR-CD3 complex: A structural and dynamical characterization of bound and unbound States. Cells. 2022;11:668. 35203317 10.3390/cells11040668 PMC8869815

[8] Jeannin P, Delneste Y, Seveso M, Life P, Bonnefoy JY. IL-12 synergizes with IL-2 and other stimuli in inducing IL-10 production by human T cells. J Immunol Baltim Md 1950. 1996;156:3159–65. 8617936

[9] Steinman RM, Cohn ZA. Identification of a novel cell type in peripheral lymphoid organs of mice. I. Morphology, quantitation, tissue distribution. J Exp Med. 1973;137:1142–62. 4573839 10.1084/jem.137.5.1142 PMC2139237

[10] Engblom C, Pfirschke C, Pittet MJ. The role of myeloid cells in cancer therapies. Nat Rev Cancer. 2016;16:447–62. 27339708 10.1038/nrc.2016.54

[11] Zhou H, Sun C, Li C, Hua S, Li F, Li R, et al. The MicroRNA-106a/20b strongly enhances the antitumour immune responses of dendritic cells pulsed with glioma stem cells by targeting STAT3. J Immunol Res. 2022;2022:9721028. 36157880 10.1155/2022/9721028 PMC9499788

[12] Yan J, Zhao Q, Gabrusiewicz K, Kong L-Y, Xia X, Wang J, et al. FGL2 promotes tumor progression in the CNS by suppressing CD103 + dendritic cell differentiation. Nat Commun. 2019;10:448. 30683885 10.1038/s41467-018-08271-x PMC6347641

[13] Osborn SL, Diehl G, Han S-J, Xue L, Kurd N, Hsieh K, et al. Fas-associated death domain (FADD) is a negative regulator of T-cell receptor-mediated necroptosis. Proc Natl Acad Sci U S A. 2010;107:13034–9. 20615958 10.1073/pnas.1005997107 PMC2919948

[14] Ji S, Yu H, Zhou D, Fan X, Duan Y, Tan Y, et al. Cancer stem cell-derived CHI3L1 activates the MAF/CTLA4 signaling pathway to promote immune escape in triple-negative breast cancer. J Transl Med. 2023;21:721. 37838657 10.1186/s12967-023-04532-6 PMC10576881

[15] Philip M, Schietinger A. CD8 + T cell differentiation and dysfunction in cancer. Nat Rev Immunol. 2022;22:209–23. 34253904 10.1038/s41577-021-00574-3 PMC9792152

[16] Heath WR, Carbone FR. Cross-presentation, dendritic cells, tolerance and immunity. Annu Rev Immunol. 2001;19:47–64. 11244030 10.1146/annurev.immunol.19.1.47

[17] Redmond WL, Marincek BC, Sherman LA. Distinct requirements for deletion versus anergy during CD8 T cell peripheral tolerance in vivo. J Immunol Baltim Md 1950. 2005;174:2046–53. 10.4049/jimmunol.174.4.2046 15699134

[18] Jhunjhunwala S, Hammer C, Delamarre L. Antigen presentation in cancer: insights into tumour immunogenicity and immune evasion. Nat Rev Cancer. 2021;21:298–312. 33750922 10.1038/s41568-021-00339-z

[19] Anderson KG, Stromnes IM, Greenberg PD. Obstacles posed by the tumor microenvironment to T cell activity: A case for synergistic therapies. Cancer Cell. 2017;31:311–25. 28292435 10.1016/j.ccell.2017.02.008 PMC5423788

[20] McKenzie B, Khazen R, Valitutti S, Greek, Fire. Poison arrows, and Scorpion bombs: how tumor cells defend against the siege weapons of cytotoxic T lymphocytes. Front Immunol. 2022;13:894306. 35592329 10.3389/fimmu.2022.894306 PMC9110820

[21] Bruni D, Angell HK, Galon J. The immune contexture and immunoscore in cancer prognosis and therapeutic efficacy. Nat Rev Cancer. 2020;20:662–80. 32753728 10.1038/s41568-020-0285-7

[22] Dunn GP, Dunn IF, Curry WT. Focus on TILs: prognostic significance of tumor infiltrating lymphocytes in human glioma. Cancer Immun. 2007;7:12. 17691714 PMC2935751

[23] Wang R, Song Y, Hu T, Wang X, Jiang Y, Zhang D, et al. Decreased CD8 + Lymphocytic infiltration in multifocal and multicentric glioblastomas. Front Oncol. 2021;11:748277. 34646781 10.3389/fonc.2021.748277 PMC8503598

[24] Han S, Zhang C, Li Q, Dong J, Liu Y, Huang Y, et al. Tumour-infiltrating CD4(+) and CD8(+) lymphocytes as predictors of clinical outcome in glioma. Br J Cancer. 2014;110:2560–8. 24691423 10.1038/bjc.2014.162 PMC4021514

[25] Kakavand H, Vilain RE, Wilmott JS, Burke H, Yearley JH, Thompson JF, et al. Tumor PD-L1 expression, immune cell correlates and PD-1 + lymphocytes in Sentinel lymph node melanoma metastases. Mod Pathol Off J U S Can Acad Pathol Inc. 2015;28:1535–44. 10.1038/modpathol.2015.110 26403784

[26] Kluger HM, Zito CR, Barr ML, Baine MK, Chiang VLS, Sznol M, et al. Characterization of PD-L1 expression and associated T-cell infiltrates in metastatic melanoma samples from variable anatomic sites. Clin Cancer Res Off J Am Assoc Cancer Res. 2015;21:3052–60. 10.1158/1078-0432.CCR-14-3073 PMC4490112 25788491

[27] Madore J, Strbenac D, Vilain R, Menzies AM, Yang JYH, Thompson JF, et al. PD-L1 negative status is associated with lower mutation burden, differential expression of Immune-Related genes, and worse survival in stage III melanoma. Clin Cancer Res Off J Am Assoc Cancer Res. 2016;22:3915–23. 10.1158/1078-0432.CCR-15-1714 26960397

[28] Obeid JM, Erdag G, Smolkin ME, Deacon DH, Patterson JW, Chen L, et al. PD-L1, PD-L2 and PD-1 expression in metastatic melanoma: correlation with tumor-infiltrating immune cells and clinical outcome. Oncoimmunology. 2016;5:e1235107. 27999753 10.1080/2162402X.2016.1235107 PMC5139635

[29] Balermpas P, Rödel F, Weiss C, Rödel C, Fokas E. Tumor-infiltrating lymphocytes favor the response to chemoradiotherapy of head and neck cancer. Oncoimmunology. 2014;3:e27403. 24711959 10.4161/onci.27403 PMC3976983

[30] de Ruiter EJ, Ooft ML, Devriese LA, Willems SM. The prognostic role of tumor infiltrating T-lymphocytes in squamous cell carcinoma of the head and neck: A systematic review and meta-analysis. Oncoimmunology. 2017;6:e1356148. 29147608 10.1080/2162402X.2017.1356148 PMC5674970

[31] Ward MJ, Thirdborough SM, Mellows T, Riley C, Harris S, Suchak K, et al. Tumour-infiltrating lymphocytes predict for outcome in HPV-positive oropharyngeal cancer. Br J Cancer. 2014;110:489–500. 24169344 10.1038/bjc.2013.639 PMC3899750

[32] Gabrielson A, Wu Y, Wang H, Jiang J, Kallakury B, Gatalica Z, et al. Intratumoral CD3 and CD8 T-cell densities associated with Relapse-Free survival in HCC. Cancer Immunol Res. 2016;4:419–30. 26968206 10.1158/2326-6066.CIR-15-0110 PMC5303359

[33] Sideras K, Biermann K, Verheij J, Takkenberg BR, Mancham S, Hansen BE, et al. PD-L1, Galectin-9 and CD8 + tumor-infiltrating lymphocytes are associated with survival in hepatocellular carcinoma. Oncoimmunology. 2017;6:e1273309. 28344887 10.1080/2162402X.2016.1273309 PMC5353918

[34] Idos GE, Kwok J, Bonthala N, Kysh L, Gruber SB, Qu C. The prognostic implications of tumor infiltrating lymphocytes in colorectal cancer: A systematic review and Meta-Analysis. Sci Rep. 2020;10:3360. 32099066 10.1038/s41598-020-60255-4 PMC7042281

[35] Alexander PG, McMillan DC, Park JH. The local inflammatory response in colorectal cancer - Type, location or density? A systematic review and meta-analysis. Cancer Treat Rev. 2020;83:101949. 31869737 10.1016/j.ctrv.2019.101949

[36] Nazemalhosseini-Mojarad E, Mohammadpour S, Torshizi Esafahani A, Gharib E, Larki P, Moradi A, et al. Intratumoral infiltrating lymphocytes correlate with improved survival in colorectal cancer patients: independent of oncogenetic features. J Cell Physiol. 2019;234:4768–77. 30370522 10.1002/jcp.27273

[37] Fridman WH, Pagès F, Sautès-Fridman C, Galon J. The immune contexture in human tumours: impact on clinical outcome. Nat Rev Cancer. 2012;12:298–306. 22419253 10.1038/nrc3245

[38] Ye S-L, Li X-Y, Zhao K, Feng T. High expression of CD8 predicts favorable prognosis in patients with lung adenocarcinoma: A cohort study. Med (Baltim). 2017;96:e6472. 10.1097/MD.0000000000006472 PMC5403074 28403077

[39] Zhang X, Kim S, Hundal J, Herndon JM, Li S, Petti AA, et al. Breast cancer neoantigens can induce CD8 + T-Cell responses and antitumor immunity. Cancer Immunol Res. 2017;5:516–23. 28619968 10.1158/2326-6066.CIR-16-0264 PMC5647648

[40] Pruneri G, Vingiani A, Denkert C. Tumor infiltrating lymphocytes in early breast cancer. Breast Edinb Scotl. 2018;37:207–14. 10.1016/j.breast.2017.03.010 28363679

[41] Aghaei M, Khademi R, Far MAJ, Bahreiny SS, Mahdizade AH, Amirrajab N. Genetic variants of dectin-1 and their antifungal immunity impact in hematologic malignancies: A comprehensive systematic review. Curr Res Transl Med. 2024;72:103460. 39038414 10.1016/j.retram.2024.103460

[42] Zhang Y, Wang X, Shi M, Song Y, Yu J, Han S. Programmed death ligand 1 and tumor-infiltrating CD8 + T lymphocytes are associated with the clinical features in meningioma. BMC Cancer. 2022;22:1171. 36371159 10.1186/s12885-022-10249-4 PMC9655806

[43] Shi M, Song Y, Zhang Y, Li L, Yu J, Hou A, et al. PD-L1 and tumor-infiltrating CD8 + lymphocytes are correlated with clinical characteristics in pediatric and adolescent pituitary adenomas. Front Endocrinol. 2023;14:1151714. 10.3389/fendo.2023.1151714 PMC10323746 37424874

[44] Shibahara I, Sonoda Y, Saito R, Kanamori M, Yamashita Y, Kumabe T, et al. The expression status of CD133 is associated with the pattern and timing of primary glioblastoma recurrence. Neuro-Oncol. 2013;15:1151–9. 23658323 10.1093/neuonc/not066 PMC3748916

[45] Ji J, Judkowski VA, Liu G, Wang H, Bunying A, Li Z, et al. Identification of novel human leukocyte antigen-A*0201-restricted, cytotoxic T lymphocyte epitopes on CD133 for cancer stem cell immunotherapy. Stem Cells Transl Med. 2014;3:356–64. 24375541 10.5966/sctm.2013-0135 PMC3952931

[46] Ricci-Vitiani L, Pallini R, Biffoni M, Todaro M, Invernici G, Cenci T, et al. Tumour vascularization via endothelial differentiation of glioblastoma stem-like cells. Nature. 2010;468:824–8. 21102434 10.1038/nature09557

[47] Beier CP, Kumar P, Meyer K, Leukel P, Bruttel V, Aschenbrenner I, et al. The cancer stem cell subtype determines immune infiltration of glioblastoma. Stem Cells Dev. 2012;21:2753–61. 22676416 10.1089/scd.2011.0660 PMC3464079

[48] Cao Y, Liu B, Cai L, Li Y, Huang Y, Zhou Y, et al. G9a promotes immune suppression by targeting the Fbxw7/Notch pathway in glioma stem cells. CNS Neurosci Ther. 2023;29:2508–21. 36971192 10.1111/cns.14191 PMC10401078

[49] Xuan W, Hsu W-H, Khan F, Dunterman M, Pang L, Wainwright DA, et al. Circadian regulator CLOCK drives immunosuppression in glioblastoma. Cancer Immunol Res. 2022;10:770–84. 35413115 10.1158/2326-6066.CIR-21-0559 PMC9177794

[50] Yuan H, Wu X, Wu Q, Chatoff A, Megill E, Gao J, et al. Lysine catabolism reprograms tumour immunity through histone crotonylation. Nature. 2023;617:818–26. 37198486 10.1038/s41586-023-06061-0 PMC11089809

[51] Plaschka M, Benboubker V, Grimont M, Berthet J, Tonon L, Lopez J, et al. ZEB1 transcription factor promotes immune escape in melanoma. J Immunother Cancer. 2022;10:e003484. 35288462 10.1136/jitc-2021-003484 PMC8921918

[52] Johansson J, Siarov J, Kiffin R, Mölne J, Mattsson J, Naredi P, et al. Presence of tumor-infiltrating CD8 + T cells and macrophages correlates to longer overall survival in patients undergoing isolated hepatic perfusion for uveal melanoma liver metastasis. Oncoimmunology. 2020;9:1854519. 33344043 10.1080/2162402X.2020.1854519 PMC7733984

[53] Yin Q, Zhao N, Chang Y, Dong M, Xu M, Xu W, et al. Melanoma stem cell vaccine induces effective tumor immunity against melanoma. Hum Vaccines Immunother. 2023;19:2158670. 10.1080/21645515.2022.2158670 PMC10114997 37067182

[54] Aikins ME, Qin Y, Dobson HE, Najafabadi AH, Lyu K, Xu Y, et al. Cancer stem cell antigen nanodisc cocktail elicits anti-tumor immune responses in melanoma. J Control Release Off J Control Release Soc. 2022;351:872–82. 10.1016/j.jconrel.2022.09.061 PMC9765445 36206945

[55] Park N, Kim KS, Na K. Stem cell-derived paracrine factors by modulated reactive oxygen species to enhance cancer immunotherapy. J Control Release Off J Control Release Soc. 2023;363:670–81. 10.1016/j.jconrel.2023.10.011 37838223

[56] Wada Y, Nakashima O, Kutami R, Yamamoto O, Kojiro M. Clinicopathological study on hepatocellular carcinoma with lymphocytic infiltration. Hepatol Baltim Md. 1998;27:407–14. 10.1002/hep.510270214 9462638

[57] Sakano Y, Noda T, Kobayashi S, Sasaki K, Iwagami Y, Yamada D, et al. Tumor endothelial cell-induced CD8 + T-cell exhaustion via GPNMB in hepatocellular carcinoma. Cancer Sci. 2022;113:1625–38. 35289033 10.1111/cas.15331 PMC9128167

[58] Xu X, Tan Y, Qian Y, Xue W, Wang Y, Du J, et al. Clinicopathologic and prognostic significance of tumor-infiltrating CD8 + T cells in patients with hepatocellular carcinoma: A meta-analysis. Med (Baltim). 2019;98:e13923. 10.1097/MD.0000000000013923 PMC6336640 30633166

[59] Miranda A, Hamilton PT, Zhang AW, Pattnaik S, Becht E, Mezheyeuski A, et al. Cancer stemness, intratumoral heterogeneity, and immune response across cancers. Proc Natl Acad Sci U S A. 2019;116:9020–9. 30996127 10.1073/pnas.1818210116 PMC6500180

[60] Wang Y-Y, Shen M-M, Gao J. Metadherin promotes stem cell phenotypes and correlated with immune infiltration in hepatocellular carcinoma. World J Gastroenterol. 2024;30:901–18. 38516242 10.3748/wjg.v30.i8.901 PMC10950638

[61] Zhu G-Q, Wang Y, Wang B, Liu W-R, Dong S-S, Chen E-B, et al. Targeting HNRNPM inhibits Cancer stemness and enhances antitumor immunity in Wnt-activated hepatocellular carcinoma. Cell Mol Gastroenterol Hepatol. 2022;13:1413–47. 35158098 10.1016/j.jcmgh.2022.02.006 PMC8938476

[62] So YK, Byeon S-J, Ku BM, Ko YH, Ahn M-J, Son Y-I, et al. An increase of CD8 + T cell infiltration following recurrence is a good prognosticator in HNSCC. Sci Rep. 2020;10:20059. 33208791 10.1038/s41598-020-77036-8 PMC7674485

[63] Sanchez-Canteli M, Granda-Díaz R, Del Rio-Ibisate N, Allonca E, López-Alvarez F, Agorreta J, et al. PD-L1 expression correlates with tumor-infiltrating lymphocytes and better prognosis in patients with HPV-negative head and neck squamous cell carcinomas. Cancer Immunol Immunother CII. 2020;69:2089–100. 32448984 10.1007/s00262-020-02604-w PMC11027666

[64] Maleki S, Schlecht NF, Keller C, Diaz J, Moss J, Prystowsky MB, et al. Lymphocytic host response to oral squamous cell carcinoma: an adaptive T-cell response at the tumor interface. Head Neck Pathol. 2011;5:117–22. 21318408 10.1007/s12105-011-0247-1 PMC3098335

[65] Soopanit T, Laokulrath N, Chayopasakul V, Pongsapich W. Prognostic value and clinicopathological status of PD-L1 expression and CD8 + TILs in oral squamous cell cancer patients with or without traditional risk factors. Head Neck. 2023;45:1017–25. 36811208 10.1002/hed.27330

[66] Aghbari SMH, Abushouk AI, Shakir OG, Zayed SO, Attia A. Correlation between tissue expression of microRNA-137 and CD8 in oral lichen planus. Clin Oral Investig. 2018;22:1463–7. 29034442 10.1007/s00784-017-2252-6

[67] Enomoto A, Sato E, Yasuda T, Isomura T, Nagao T, Chikazu D. Intraepithelial CD8 + lymphocytes as a predictive diagnostic biomarker for the remission of oral lichen planus. Hum Pathol. 2018;74:43–53. 29288692 10.1016/j.humpath.2017.12.008

[68] Öhman J, Mowjood R, Larsson L, Kovacs A, Magnusson B, Kjeller G, et al. Presence of CD3-positive T-cells in oral premalignant leukoplakia indicates prevention of cancer transformation. Anticancer Res. 2015;35:311–7. 25550565

[69] Chaves ALF, Silva AG, Maia FM, Lopes GFM, De Paulo LFB, Muniz LV, et al. Reduced CD8 + T cells infiltration can be associated to a malignant transformation in potentially malignant oral epithelial lesions. Clin Oral Investig. 2019;23:1913–9. 30229300 10.1007/s00784-018-2622-8

[70] De Meulenaere A, Vermassen T, Aspeslagh S, Deron P, Duprez F, Laukens D, et al. Tumor PD-L1 status and CD8 + tumor-infiltrating T cells: markers of improved prognosis in oropharyngeal cancer. Oncotarget. 2017;8:80443–52. 29113315 10.18632/oncotarget.19045 PMC5655210

[71] Ma N, Hua R, Yang Y, Liu Z-C, Pan J, Yu B-Y, et al. PES1 reduces CD8 + T cell infiltration and immunotherapy sensitivity via interrupting ILF3-IL15 complex in esophageal squamous cell carcinoma. J Biomed Sci. 2023;30:20. 36959575 10.1186/s12929-023-00912-8 PMC10037800

[72] Tapias LF, Shih A, Mino-Kenudson M, Muniappan A, Gaissert HA, Lanuti M, et al. Programmed death ligand 1 and CD8 + immune cell infiltrates in resected primary tracheal malignant neoplasms. Eur J Cardiothorac Surg. 2019;55:691–8. 30418532 10.1093/ejcts/ezy370

[73] Qin Z, Zhang W, Liu S, Wang Y, Peng X, Jia L. PVT1 Inhibition stimulates anti-tumor immunity, prevents metastasis, and depletes cancer stem cells in squamous cell carcinoma. Cell Death Dis. 2023;14:187. 36894542 10.1038/s41419-023-05710-6 PMC9998619

[74] Wang C, Li Y, Jia L, Kim JK, Li J, Deng P, et al. CD276 expression enables squamous cell carcinoma stem cells to evade immune surveillance. Cell Stem Cell. 2021;28:1597–e16137. 33945793 10.1016/j.stem.2021.04.011 PMC8419062

[75] Liu S, Qin Z, Mao Y, Wang N, Zhang W, Wang Y, et al. Pharmacological Inhibition of MYC to mitigate chemoresistance in preclinical models of squamous cell carcinoma. Theranostics. 2024;14:622–39. 38169606 10.7150/thno.88759 PMC10758066

[76] Lequerica-Fernández P, Suárez-Canto J, Rodriguez-Santamarta T, Rodrigo JP, Suárez-Sánchez FJ, Blanco-Lorenzo V, et al. Prognostic relevance of CD4+, CD8 + and FOXP3 + TILs in oral squamous cell carcinoma and correlations with PD-L1 and Cancer stem cell markers. Biomedicines. 2021;9:653. 34201050 10.3390/biomedicines9060653 PMC8227658

[77] Xu Y, Dong X, Qi P, Ye Y, Shen W, Leng L, et al. Sox2 communicates with Tregs through CCL1 to promote the stemness property of breast Cancer cells. Stem Cells Dayt Ohio. 2017;35:2351–65. 10.1002/stem.2720 PMC5958902 29044882

[78] Li D, Zhao W, Zhang X, Lv H, Li C, Sun L. NEFM DNA methylation correlates with immune infiltration and survival in breast cancer. Clin Epigenetics. 2021;13:112. 34001208 10.1186/s13148-021-01096-4 PMC8130356

[79] De Groot AF, Blok EJ, Charehbili A, Engels CC, Smit VTHBM, Dekker-Ensink NG, et al. Strong CD8 + lymphocyte infiltration in combination with expression of HLA class I is associated with better tumor control in breast cancer patients treated with neoadjuvant chemotherapy. Breast Cancer Res Treat. 2019;175:605–15. 30868392 10.1007/s10549-019-05195-y PMC6534526

[80] Jamiyan T, Kuroda H, Yamaguchi R, Nakazato Y, Noda S, Onozaki M, et al. Prognostic impact of a tumor-infiltrating lymphocyte subtype in triple negative cancer of the breast. Breast Cancer. 2020;27:880–92. 32222891 10.1007/s12282-020-01084-1 PMC7438376

[81] Quaglino E, Conti L, Cavallo F. Breast cancer stem cell antigens as targets for immunotherapy. Semin Immunol. 2020;47:101386. 31932198 10.1016/j.smim.2020.101386

[82] Tay AS-MS, Amano T, Edwards LA, Yu JS. CD133 mRNA-transfected dendritic cells induce coordinated cytotoxic and helper T cell responses against breast cancer stem cells. Mol Ther Oncolytics. 2021;22:64–71. 34485687 10.1016/j.omto.2021.05.006 PMC8403713

[83] Gao W, Wen H, Liang L, Dong X, Du R, Zhou W, et al. IL20RA signaling enhances stemness and promotes the formation of an immunosuppressive microenvironment in breast cancer. Theranostics. 2021;11:2564–80. 33456560 10.7150/thno.45280 PMC7806486

[84] Chen C, Bai L, Cao F, Wang S, He H, Song M, et al. Targeting LIN28B reprograms tumor glucose metabolism and acidic microenvironment to suppress cancer stemness and metastasis. Oncogene. 2019;38:4527–39. 30742065 10.1038/s41388-019-0735-4

[85] Qi M, Xia Y, Wu Y, Zhang Z, Wang X, Lu L, et al. Lin28B-high breast cancer cells promote immune suppression in the lung pre-metastatic niche via exosomes and support cancer progression. Nat Commun. 2022;13:897. 35173168 10.1038/s41467-022-28438-x PMC8850492

[86] Liu H, Yan R, Xiao Z, Huang X, Yao J, Liu J, et al. Targeting DCLK1 attenuates tumor stemness and evokes antitumor immunity in triple-negative breast cancer by inhibiting IL-6/STAT3 signaling. Breast Cancer Res BCR. 2023;25:43. 37069669 10.1186/s13058-023-01642-3 PMC10108533

[87] Zheng H, Ning Y, Yang Y, Zhan Y, Wang H, Wen Q, et al. Aberrant expression of β-Catenin correlates with infiltrating immune cells and prognosis in NSCLC. Pathol Oncol Res. 2021;27:1609981. 34764821 10.3389/pore.2021.1609981 PMC8575687

[88] Henry CJ, Ornelles DA, Mitchell LM, Brzoza-Lewis KL, Hiltbold EM. IL-12 produced by dendritic cells augments CD8 + T cell activation through the production of the chemokines CCL1 and CCL17. J Immunol Baltim Md 1950. 2008;181:8576–84. 10.4049/jimmunol.181.12.8576 PMC2716729 19050277

[89] Semmling V, Lukacs-Kornek V, Thaiss CA, Quast T, Hochheiser K, Panzer U, et al. Alternative cross-priming through CCL17-CCR4-mediated attraction of CTLs toward NKT cell-licensed DCs. Nat Immunol. 2010;11:313–20. 20190758 10.1038/ni.1848

[90] Li Y, Zhu L, Hao R, Li Y, Zhao Q, Li S. Systematic expression analysis of the CELSR family reveals the importance of CELSR3 in human lung adenocarcinoma. J Cell Mol Med. 2021;25:4349–62. 33811453 10.1111/jcmm.16497 PMC8093986

[91] Corgnac S, Damei I, Gros G, Caidi A, Terry S, Chouaib S, et al. Cancer stem-like cells evade CD8 + CD103 + tumor-resident memory T (TRM) lymphocytes by initiating an epithelial-to-mesenchymal transition program in a human lung tumor model. J Immunother Cancer. 2022;10:e004527. 35418483 10.1136/jitc-2022-004527 PMC9014106

[92] Hong W, Xue M, Jiang J, Zhang Y, Gao X. Circular RNA circ-CPA4/ let-7 miRNA/PD-L1 axis regulates cell growth, stemness, drug resistance and immune evasion in non-small cell lung cancer (NSCLC). J Exp Clin Cancer Res CR. 2020;39:149. 32746878 10.1186/s13046-020-01648-1 PMC7397626

[93] Kursunel MA, Taskiran EZ, Tavukcuoglu E, Yanik H, Demirag F, Karaosmanoglu B, et al. Small cell lung cancer stem cells display mesenchymal properties and exploit immune checkpoint pathways in activated cytotoxic T lymphocytes. Cancer Immunol Immunother CII. 2022;71:445–59. 34228218 10.1007/s00262-021-02998-1 PMC8783896

[94] Pagès F, Mlecnik B, Marliot F, Bindea G, Ou F-S, Bifulco C, et al. International validation of the consensus immunoscore for the classification of colon cancer: a prognostic and accuracy study. Lancet Lond Engl. 2018;391:2128–39. 10.1016/S0140-6736(18)30789-X 29754777

[95] Zhao Y, Ge X, He J, Cheng Y, Wang Z, Wang J, et al. The prognostic value of tumor-infiltrating lymphocytes in colorectal cancer differs by anatomical subsite: a systematic review and meta-analysis. World J Surg Oncol. 2019;17:85. 31118034 10.1186/s12957-019-1621-9 PMC6532263

[96] Zhang S, Zhong M, Wang C, Xu Y, Gao W-Q, Zhang Y. CCL5-deficiency enhances intratumoral infiltration of CD8 + T cells in colorectal cancer. Cell Death Dis. 2018;9:766. 29991744 10.1038/s41419-018-0796-2 PMC6039518

[97] Xue J, Yu X, Xue L, Ge X, Zhao W, Peng W. Intrinsic β-catenin signaling suppresses CD8 + T-cell infiltration in colorectal cancer. Biomed Pharmacother. 2019;115:108921. 31078045 10.1016/j.biopha.2019.108921

[98] Noh B-J, Kwak JY, Eom D-W. Immune classification for the PD-L1 expression and tumour-infiltrating lymphocytes in colorectal adenocarcinoma. BMC Cancer. 2020;20:58. 31992245 10.1186/s12885-020-6553-9 PMC6986059

[99] Shang S, Yang C, Chen F, Xiang R-S, Zhang H, Dai S-Y, et al. ID1 expressing macrophages support cancer cell stemness and limit CD8 + T cell infiltration in colorectal cancer. Nat Commun. 2023;14:7661. 37996458 10.1038/s41467-023-43548-w PMC10667515

[100] Mennonna D, Maccalli C, Romano MC, Garavaglia C, Capocefalo F, Bordoni R, et al. T cell neoepitope discovery in colorectal cancer by high throughput profiling of somatic mutations in expressed genes. Gut. 2017;66:454–63. 26681737 10.1136/gutjnl-2015-309453 PMC5534766

[101] Miyamoto S, Kochin V, Kanaseki T, Hongo A, Tokita S, Kikuchi Y, et al. The antigen ASB4 on Cancer stem cells serves as a target for CTL immunotherapy of colorectal Cancer. Cancer Immunol Res. 2018;6:358–69. 29371260 10.1158/2326-6066.CIR-17-0518

[102] Lin H, Wei S, Hurt EM, Green MD, Zhao L, Vatan L, et al. Host expression of PD-L1 determines efficacy of PD-L1 pathway blockade-mediated tumor regression. J Clin Invest. 2018;128:805–15. 29337305 10.1172/JCI96113 PMC5785251

[103] Yang L, Shi P, Zhao G, Xu J, Peng W, Zhang J, et al. Targeting cancer stem cell pathways for cancer therapy. Signal Transduct Target Ther. 2020;5:8. 32296030 10.1038/s41392-020-0110-5 PMC7005297

[104] Zhao L, Cheng S, Fan L, Zhang B, Xu S. TIM-3: an update on immunotherapy. Int Immunopharmacol. 2021;99:107933. 34224993 10.1016/j.intimp.2021.107933

[105] Chocarro L, Blanco E, Zuazo M, Arasanz H, Bocanegra A, Fernández-Rubio L, et al. Understanding LAG-3 signaling. Int J Mol Sci. 2021;22:5282. 34067904 10.3390/ijms22105282 PMC8156499

[106] Li M, Knight DA, Smyth MJ, Stewart TJ. Sensitivity of a novel model of mammary cancer stem cell-like cells to TNF-related death pathways. Cancer Immunol Immunother CII. 2012;61:1255–68. 22270714 10.1007/s00262-012-1200-1 PMC11029674

[107] Liu L, Wang A, Liu X, Han S, Sun Y, Zhang J, et al. Blocking TIGIT/CD155 signalling reverses CD8 + T cell exhaustion and enhances the antitumor activity in cervical cancer. J Transl Med. 2022;20:280. 35729552 10.1186/s12967-022-03480-x PMC9210727

[108] Hassn Mesrati M, Syafruddin SE, Mohtar MA, Syahir A. CD44: A multifunctional mediator of Cancer progression. Biomolecules. 2021;11:1850. 34944493 10.3390/biom11121850 PMC8699317

[109] Bhummaphan N, Petpiroon N, Prakhongcheep O, Sritularak B, Chanvorachote P. Lusianthridin targeting of lung cancer stem cells via Src-STAT3 suppression. Phytomedicine Int J Phytother Phytopharm. 2019;62:152932. 10.1016/j.phymed.2019.152932 31100681

[110] Feng Y, Cai L, Pook M, Liu F, Chang C-H, Mouti MA, et al. BRD9-SMAD2/3 orchestrates stemness and tumorigenesis in pancreatic ductal adenocarcinoma. Gastroenterology. 2024;166:139–54. 37739089 10.1053/j.gastro.2023.09.021 PMC11304550

[111] Xie F, Zhou X, Su P, Li H, Tu Y, Du J, et al. Breast cancer cell-derived extracellular vesicles promote CD8 + T cell exhaustion via TGF-β type II receptor signaling. Nat Commun. 2022;13:4461. 35915084 10.1038/s41467-022-31250-2 PMC9343611

[112] Mittendorf EA, Philips AV, Meric-Bernstam F, Qiao N, Wu Y, Harrington S, et al. PD-L1 expression in triple-negative breast cancer. Cancer Immunol Res. 2014;2:361–70. 24764583 10.1158/2326-6066.CIR-13-0127 PMC4000553

[113] Szarynska M, Olejniczak A, Wierzbicki P, Kobiela J, Laski D, Sledzinski Z, et al. FasR and FasL in colorectal cancer. Int J Oncol. 2017;51:975–86. 28766682 10.3892/ijo.2017.4083

[114] Alahdal M, Xing Y, Tang T, Liang J. 1-Methyl-D-tryptophan reduces tumor CD133 + cells, Wnt/β-catenin and NF-κβp65 while enhances lymphocytes NF-κβ2, STAT3, and STAT4 pathways in murine pancreatic adenocarcinoma. Sci Rep. 2018;8:9869. 29959375 10.1038/s41598-018-28238-8 PMC6026162

[115] Rashidi A, Miska J, Lee-Chang C, Kanojia D, Panek WK, Lopez-Rosas A, et al. GCN2 is essential for CD8 + T cell survival and function in murine models of malignant glioma. Cancer Immunol Immunother CII. 2020;69:81–94. 31844909 10.1007/s00262-019-02441-6 PMC6952559

[116] Misra J, Holmes MJ, Mirek T, Langevin E, Kim M, Carlson H-G. Discordant regulation of eIF2 kinase GCN2 and mTORC1 during nutrient stress. Nucleic Acids Res. 2021;49:5726–42. 34023907 10.1093/nar/gkab362 PMC8191763

[117] Liu Y, Liang X, Dong W, Fang Y, Lv J, Zhang T, et al. Tumor-Repopulating cells induce PD-1 expression in CD8 + T cells by transferring kynurenine and AhR activation. Cancer Cell. 2018;33:480–e4947. 29533786 10.1016/j.ccell.2018.02.005

[118] Sancho P, Barneda D, Heeschen C. Hallmarks of cancer stem cell metabolism. Br J Cancer. 2016;114:1305–12. 27219018 10.1038/bjc.2016.152 PMC4984474

[119] Kumagai S, Koyama S, Itahashi K, Tanegashima T, Lin Y-T, Togashi Y, et al. Lactic acid promotes PD-1 expression in regulatory T cells in highly glycolytic tumor microenvironments. Cancer Cell. 2022;40:201–e2189. 35090594 10.1016/j.ccell.2022.01.001

[120] Xiong Z, Chan SL, Zhou J, Vong JSL, Kwong TT, Zeng X, et al. Targeting PPAR-gamma counteracts tumour adaptation to immune-checkpoint Blockade in hepatocellular carcinoma. Gut. 2023;72:1758–73. 37019619 10.1136/gutjnl-2022-328364 PMC10423534

[121] Aghapour SA, Torabizadeh M, Bahreiny SS, Saki N, Jalali Far MA, Yousefi-Avarvand A, et al. Investigating the dynamic interplay between cellular immunity and tumor cells in the fight against cancer: an updated comprehensive review. Iran J Blood Cancer. 2024;16:84–101.

[122] Solis-Castillo LA, Garcia-Romo GS, Diaz-Rodriguez A, Reyes-Hernandez D, Tellez-Rivera E, Rosales-Garcia VH, et al. Tumor-infiltrating regulatory T cells, CD8/Treg ratio, and cancer stem cells are correlated with lymph node metastasis in patients with early breast cancer. Breast Cancer Tokyo Jpn. 2020;27:837–49. 10.1007/s12282-020-01079-y 32180141

[123] Napoletano C, Bellati F, Ruscito I, Pernice M, Zizzari IG, Caponnetto S, et al. Immunological and clinical impact of Cancer stem cells in vulvar cancer: role of CD133/CD24/ABCG2-Expressing cells. Anticancer Res. 2016;36:5109–16. 27798870 10.21873/anticanres.11080

[124] Wei J, Wu A, Kong L-Y, Wang Y, Fuller G, Fokt I, et al. Hypoxia potentiates glioma-mediated immunosuppression. PLoS ONE. 2011;6:e16195. 21283755 10.1371/journal.pone.0016195 PMC3024401

[125] Yu X, Li H, Ren X. Interaction between regulatory T cells and cancer stem cells. Int J Cancer. 2012;131:1491–8. 22592629 10.1002/ijc.27634

[126] Welte T, Kim IS, Tian L, Gao X, Wang H, Li J, et al. Oncogenic mTOR signalling recruits myeloid-derived suppressor cells to promote tumour initiation. Nat Cell Biol. 2016;18:632–44. 27183469 10.1038/ncb3355 PMC4884142

[127] Peng D, Tanikawa T, Li W, Zhao L, Vatan L, Szeliga W, et al. Myeloid-Derived suppressor cells endow Stem-like qualities to breast Cancer cells through IL6/STAT3 and NO/NOTCH Cross-talk signaling. Cancer Res. 2016;76:3156–65. 27197152 10.1158/0008-5472.CAN-15-2528 PMC4891237

[128] Xie M, Lin Z, Ji X, Luo X, Zhang Z, Sun M, et al. FGF19/FGFR4-mediated elevation of ETV4 facilitates hepatocellular carcinoma metastasis by upregulating PD-L1 and CCL2. J Hepatol. 2023;79:109–25. 36907560 10.1016/j.jhep.2023.02.036

[129] Lei MML, Lee TKW. Cancer stem cells: emerging key players in immune evasion of cancers. Front Cell Dev Biol. 2021;9:692940. 34235155 10.3389/fcell.2021.692940 PMC8257022

[130] Yang L, Zhang Y. Tumor-associated macrophages, potential targets for cancer treatment. Biomark Res. 2017;5:25. 28804638 10.1186/s40364-017-0106-7 PMC5549387

[131] Whiteside TL. The tumor microenvironment and its role in promoting tumor growth. Oncogene. 2008;27:5904–12. 18836471 10.1038/onc.2008.271 PMC3689267

[132] Martinez FO, Sica A, Mantovani A, Locati M. Macrophage activation and polarization. Front Biosci J Virtual Libr. 2008;13:453–61. 10.2741/2692 17981560

[133] Ramos KS, Montoya-Durango DE, Teneng I, Nanez A, Stribinskis V. Epigenetic control of embryonic renal cell differentiation by L1 retrotransposon. Birt Defects Res Clin Mol Teratol. 2011;91:693–702. 10.1002/bdra.20786 PMC3180906 21384534

[134] Mohamed Ariff I, Mitra A, Basu A. Epigenetic regulation of self-renewal and fate determination in neural stem cells. J Neurosci Res. 2012;90:529–39. 22183977 10.1002/jnr.22804

[135] Pathania R, Ramachandran S, Mariappan G, Thakur P, Shi H, Choi J-H, et al. Combined Inhibition of DNMT and HDAC blocks the tumorigenicity of Cancer Stem-like cells and attenuates mammary tumor growth. Cancer Res. 2016;76:3224–35. 27197203 10.1158/0008-5472.CAN-15-2249 PMC4891240

[136] Sadelain M, Rivière I, Brentjens R. Targeting tumours with genetically enhanced T lymphocytes. Nat Rev Cancer. 2003;3:35–45. 12509765 10.1038/nrc971

[137] Sterner RC, Sterner RM. CAR-T cell therapy: current limitations and potential strategies. Blood Cancer J. 2021;11:69. 33824268 10.1038/s41408-021-00459-7 PMC8024391

[138] Drent E, Groen RWJ, Noort WA, Themeli M, van Lammerts JJ, Parren PWHI, et al. Pre-clinical evaluation of CD38 chimeric antigen receptor engineered T cells for the treatment of multiple myeloma. Haematologica. 2016;101:616–25. 26858358 10.3324/haematol.2015.137620 PMC5004365

[139] Laborda E, Mazagova M, Shao S, Wang X, Quirino H, Woods AK, et al. Development of A chimeric antigen receptor targeting C-Type Lectin-Like Molecule-1 for human acute myeloid leukemia. Int J Mol Sci. 2017;18:2259. 29077054 10.3390/ijms18112259 PMC5713229

[140] Huang J, Yang Y, Fang F, Liu K. MALAT1 modulates the autophagy of retinoblastoma cell through miR-124-mediated stx17 regulation. J Cell Biochem. 2018;119:3853–63. 29073720 10.1002/jcb.26464

[141] Drent E, Poels R, Ruiter R, van de Donk NWCJ, Zweegman S, Yuan H, et al. Combined CD28 and 4-1BB costimulation potentiates Affinity-tuned chimeric antigen Receptor-engineered T cells. Clin Cancer Res Off J Am Assoc Cancer Res. 2019;25:4014–25. 10.1158/1078-0432.CCR-18-2559 PMC7477921 30979735

[142] Masoumi J, Jafarzadeh A, Abdolalizadeh J, Khan H, Philippe J, Mirzaei H, et al. Cancer stem cell-targeted chimeric antigen receptor (CAR)-T cell therapy: challenges and prospects. Acta Pharm Sin B. 2021;11:1721–39. 34386318 10.1016/j.apsb.2020.12.015 PMC8343118

[143] Zhao Y, Fei Y, Zhao Y, Li M, Hu Y, Cai K, et al. Biomineralization-Tuned nanounits reprogram the signal transducer and activator of transcription 3 signaling for Ferroptosis-Immunotherapy in Cancer stem cells. ACS Nano. 2024;18:21268–87. 39083438 10.1021/acsnano.4c05084

[144] Saki N, Haybar H, Aghaei M, Subject. Motivation can be suppressed, but scientific ability cannot and should not be ignored. J Transl Med. 2023;21:520. 37533088 10.1186/s12967-023-04383-1 PMC10398917

[145] Aghaei M, Khademi R, Bahreiny SS, Saki N. The need to Establish and recognize the field of clinical laboratory science (CLS) as an essential field in advancing clinical goals. Health Sci Rep. 2024;7:e70008. 39170888 10.1002/hsr2.70008 PMC11335574

[146] Dai H, Tong C, Shi D, Chen M, Guo Y, Chen D, et al. Efficacy and biomarker analysis of CD133-directed CAR T cells in advanced hepatocellular carcinoma: a single-arm, open-label, phase II trial. Oncoimmunology. 2020;9:1846926. 33312759 10.1080/2162402X.2020.1846926 PMC7714531

[147] Correction. GB1275, a first-in-class CD11b modulator: rationale for immunotherapeutic combinations in solid tumors. J Immunother Cancer. 2021;9:e003005corr1. 34725217 10.1136/jitc-2021-003005corr1 PMC8562507

[148] Li Q, Han J, Yang Y, Chen Y. PD-1/PD-L1 checkpoint inhibitors in advanced hepatocellular carcinoma immunotherapy. Front Immunol. 2022;13:1070961. 36601120 10.3389/fimmu.2022.1070961 PMC9806143

[149] Klement JD, Paschall AV, Redd PS, Ibrahim ML, Lu C, Yang D, et al. An osteopontin/CD44 immune checkpoint controls CD8 + T cell activation and tumor immune evasion. J Clin Invest. 2018;128:5549–60. 30395540 10.1172/JCI123360 PMC6264631

[150] Yang C, You J, Pan Q, Tang Y, Cai L, Huang Y, et al. Targeted delivery of a PD-1-blocking ScFv by CD133-specific CAR-T cells using nonviral sleeping beauty transposition shows enhanced antitumour efficacy for advanced hepatocellular carcinoma. BMC Med. 2023;21:327. 37635247 10.1186/s12916-023-03016-0 PMC10464109

[151] Dahmani A, Delisle J-S. TGF-β in T cell biology: implications for Cancer immunotherapy. Cancers. 2018;10:194. 29891791 10.3390/cancers10060194 PMC6025055

[152] Sullivan KM, Jiang X, Guha P, Lausted C, Carter JA, Hsu C, et al. Blockade of Interleukin 10 potentiates antitumour immune function in human colorectal cancer liver metastases. Gut. 2023;72:325–37. 35705369 10.1136/gutjnl-2021-325808 PMC9872249

[153] Yang C, Wu S, Mou Z, Zhou Q, Dai X, Ou Y, et al. Exosome-derived circTRPS1 promotes malignant phenotype and CD8 + T cell exhaustion in bladder cancer microenvironments. Mol Ther J Am Soc Gene Ther. 2022;30:1054–70. 10.1016/j.ymthe.2022.01.022 PMC8899700 35038580

[154] Liang X, Gao H, Xiao J, Han S, He J, Yuan R, et al. Abrine, an IDO1 inhibitor, suppresses the immune escape and enhances the immunotherapy of anti-PD-1 antibody in hepatocellular carcinoma. Front Immunol. 2023;14:1185985. 37334368 10.3389/fimmu.2023.1185985 PMC10272936

[155] Babl N, Decking S-M, Voll F, Althammer M, Sala-Hojman A, Ferretti R, et al. MCT4 Blockade increases the efficacy of immune checkpoint Blockade. J Immunother Cancer. 2023;11:e007349. 37880183 10.1136/jitc-2023-007349 PMC10603342

[156] Pelly VS, Moeini A, Roelofsen LM, Bonavita E, Bell CR, Hutton C, et al. Anti-Inflammatory drugs remodel the tumor immune environment to enhance immune checkpoint Blockade efficacy. Cancer Discov. 2021;11:2602–19. 34031121 10.1158/2159-8290.CD-20-1815 PMC7611767

[157] Lin S, Zhang A, Yuan L, Wang Y, Zhang C, Jiang J, et al. Targeting parvalbumin promotes M2 macrophage polarization and energy expenditure in mice. Nat Commun. 2022;13:3301. 35676256 10.1038/s41467-022-30757-y PMC9177846

[158] Pathania R, Ramachandran S, Elangovan S, Padia R, Yang P, Cinghu S, et al. DNMT1 is essential for mammary and cancer stem cell maintenance and tumorigenesis. Nat Commun. 2015;6:6910. 25908435 10.1038/ncomms7910 PMC4410389

[159] Bernal-Estévez DA, Ortíz Barbosa MA, Ortíz-Montero P, Cifuentes C, Sánchez R, Parra-López CA. Autologous dendritic cells in combination with chemotherapy restore responsiveness of T cells in breast Cancer patients: A Single-Arm phase I/II trial. Front Immunol. 2021;12:669965. 34489928 10.3389/fimmu.2021.669965 PMC8417880

